# Reduced-Dose Hypofractionated Radiation Therapy (3 Gy × 3 Fractions) for Indolent Non-Hodgkin’s lymphoma (POSEIDON): A Multisite Phase 2 Randomized Trial Protocol

**DOI:** 10.1016/j.adro.2025.101908

**Published:** 2025-09-26

**Authors:** Omran Saifi, Scott C. Lester, William G. Rule, William Breen, Randa Tao, Jason R. Young, Liuyan Jiang, Han W. Tun, Emily Liu, Lauren E. Haydu, Allison Rosenthal, Javier Munoz, Jose Caetano Villasboas, Yucai Wang, Muhamad Alhaj Moustafa, Madiha Iqbal, Anna C. Harrell, Jennifer L. Peterson, Bradford S. Hoppe

**Affiliations:** a Department of Radiation Oncology, Mayo Clinic, Jacksonville, Florida; b Department of Radiation Oncology, Mayo Clinic, Rochester, Minnesota; c Department of Radiation Oncology, Mayo Clinic, Phoenix, Arizona; d Departments of Radiology, Mayo Clinic, Jacksonville, Florida; e Departments of Pathology, Mayo Clinic, Jacksonville, Florida; f Division of Hematology, Mayo Clinic, Jacksonville, Florida; g Division of Clinical Trials and Biostatistics, Mayo Clinic, Jacksonville, Florida; h Division of Hematology, Mayo Clinic, Phoenix, Arizona; i Division of Hematology, Mayo Clinic, Rochester, Minnesota

## Abstract

Indolent non-Hodgkin’s lymphoma, including follicular and marginal zone lymphoma, is highly radiosensitive, with radiation therapy (RT) serving as an effective treatment. Although standard RT doses (24 Gy in 12 fractions) provide excellent disease control, they are associated with toxicity. Emerging evidence suggests that lower RT doses may maintain efficacy while reducing toxicity; however, prior prospective randomized attempts to reduce the dose to 4 Gy in 2 fractions have demonstrated inferior disease control. This phase 2 randomized trial aims to determine whether reduced-dose hypofractionated RT can achieve comparable disease control while minimizing toxicity and treatment burden. Patients will be randomized 1:1 to receive experimental arm treatment with 8 to 10 Gy in 2 to 5 fractions or standard of care treatment with 24 Gy in 12 fractions. The primary endpoint is acute toxicity (grade ≥ 2). Secondary endpoints include patient-reported quality of life (FACIT-Fatigue scale), response rate at 3 months posttreatment (Lugano criteria), local control, relapse-free survival, and overall survival. Exploratory analyses will evaluate financial toxicity (COST-FACIT questionnaire), health care expenditure, late toxicity, and the prognostic value of preradiation metabolic imaging parameters, including metabolic tumor volume, total lesion glycolysis, and maximum standardized uptake value, as well as molecular biomarkers such as *TP53, MYC*, and Ki-67.

## Introduction

Studies evaluating the use of radiation therapy (RT) in patients with early-stage indolent lymphoma have typically used radiation doses between 20 Gy and 50 Gy. Prospective studies have demonstrated that lower radiation doses (24 Gy) appeared to be as effective as higher doses (45-50 Gy).^1^

Furthermore, retrospective and single-arm prospective studies showed that 4 Gy in 2 fractions achieved high response rate with minimal toxic effects, much shorter treatment course, and lower cost.^2^, ^3^, ^4^ Unfortunately, prospective attempts to replicate this reduction of RT dose for follicular and marginal zone lymphoma showed inferior disease control.^5^ The Follicular Radiotherapy Trial (FoRT) study showed a 2-year local progression-free rate of 94.1% after 24 Gy and 79.8% after 4 Gy confirming 24 Gy as the standard of care. However, toxicity rates were significantly worse with 24 Gy compared to 4 Gy (grade 2+ acute toxicity: 34% vs 10%),^5^ which have led many to continue to use 4 Gy despite the inferior local control.

Currently, risk stratification for indolent lymphoma includes follicular lymphoma international prognostic index and follicular lymphoma international prognostic index 2, which use traditional prognostic factors based on blood tests (Lactate Dehydrogenase (LDH), beta microglobulin) and computed tomography imaging for size and sites involved, but do not take into consideration more contemporary techniques, such as functional imaging segmentation values and molecular classification.

The purpose of this prospective project is to evaluate reduction of RT dose for patients with indolent non-Hodgkin’s lymphoma (NHL) by conducting a prospective randomized phase 2 study (POSEIDON) comparing reduced-dose hypofractionated involved-site RT of 8 to 10 Gy delivered over 2 to 5 fractions (biologically effective dose, 11-12 Gy) to standard of care 24 Gy delivered over 12 fractions (biologically effective dose, 29 Gy), while simultaneously evaluating prognostic value of preradiation functional imaging parameters and molecular markers to predict treatment response and failure.

## Research Methods and Analysis

**Figure 1:**
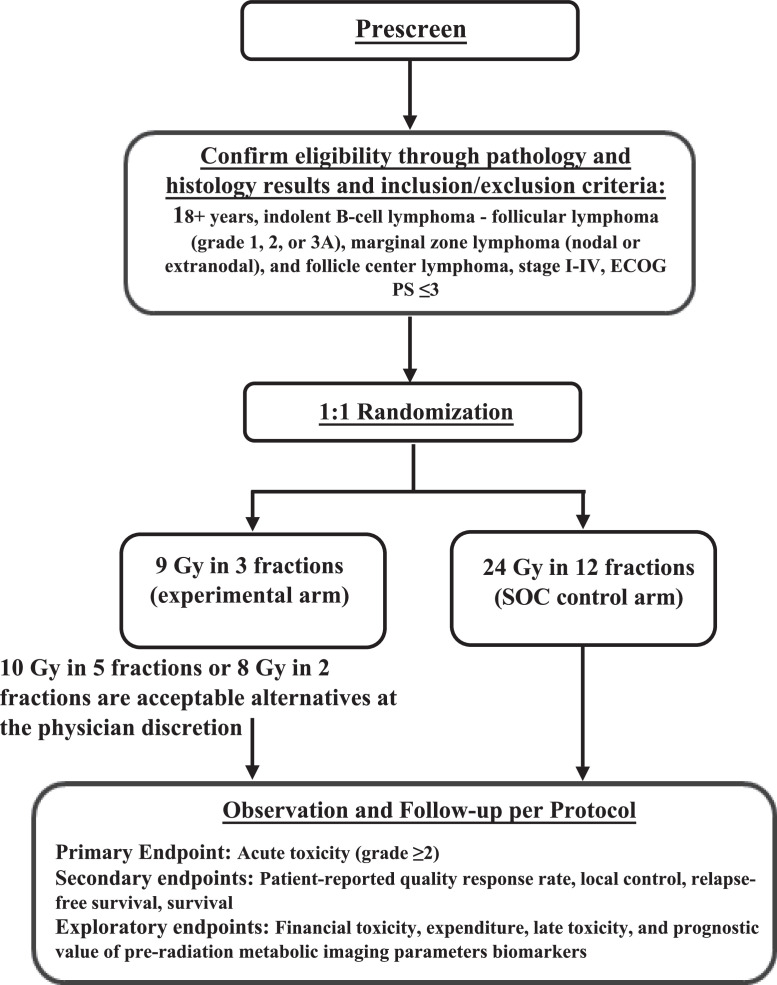
Protocol schema of the POSEIDON trial.

The study is designed as a 2-arm 1:1 randomized phase 2 trial to evaluate an intermediate dose of 8 to 10 Gy delivered over 2 to 5 fractions versus the standard of care (SOC) 24 Gy in 12 fractions for patients with indolent lymphoma, while simultaneously evaluating prognostic value of preradiation functional imaging parameters and molecular characteristics to predict treatment response and failure (Fig. 1).

The primary goal of this randomized phase 2 trial is to demonstrate that the experimental arm, which includes 9 Gy in 3 fractions, 8 Gy in 2 fractions,^6^ or 10 Gy in 5 fractions, significantly reduces acute toxicity compared to the standard 24 Gy in 12 fractions. Acute toxicity, defined as grade ≥ 2 adverse events at least possibly related to radiation treatment, will be assessed at the end of treatment, 7 days postradiation, and 14 days postradiation, according to Common Terminology Criteria for Adverse Events (CTCAE) version 5 criteria.

The secondary goals of the trial focus on several key outcomes. Patient-reported quality of life will be assessed using the FACIT-Fatigue scale. The response rate will be evaluated at 3 months post radiation treatment according to Lugano criteria.^7^ The local control rate will be monitored up to 2 years after radiation treatment, defined as the time from registration to local relapse within the treated site (95% isodose line). Additionally, relapse-free survival, defined as the time from registration to any local or distant relapse, and overall survival, defined as the time from registration to death from any cause, will be tracked for up to 24 months after the completion of radiation treatment.

Exploratory goals of the study include evaluating financial toxicity and health care expenditure. Financial toxicity will be measured using a modified COST-FACIT questionnaire, while health care expenditure will reflect the costs associated with cancer treatment and related activities. Additionally, late toxicity, defined as adverse events at least possibly related to radiation treatment, will be evaluated at 3 months following treatment completion.

Correlative research will use diagnostic biopsies obtained at the time of initial diagnosis. No new biopsies will be collected. If retrievable, these archival specimens will be analyzed for molecular and immunohistochemical markers potentially associated with disease relapse and radiation resistance. This includes evaluating *TP53* gene alterations through fluorescence in situ hybridization, as well as the expression of *TP53, MYC*, and the proliferation index (Ki-67) using immunohistochemistry staining. Archived tissue from patient biopsies will also be analyzed for cellular and genetic mutations linked to disease relapse and radiation resistance. Baseline positron emission tomography/computed tomography scans of patients will be used to calculate metabolic tumor volume, total lesion glycolysis, and maximum standardized uptake value, which will then be correlated with disease relapse and treatment response.

Eligibility criteria include being aged 18 years or older and having histologically confirmed indolent B-cell lymphoma, including subtypes such as follicular lymphoma (grade 1, 2, or 3A), marginal zone lymphoma (nodal or extranodal), and follicle center lymphoma. Patients with any stage of disease, whether initial, refractory, or relapsed, are eligible, provided that if relapse involves the site to be treated, there is evidence of disease progression. An Eastern Cooperative Oncology Group performance status of ≤3 is required. For individuals of childbearing potential, a negative pregnancy test must be conducted within 7 days prior to registration. Additionally, participants must be confirmed by a radiation oncologist as suitable for participation in the study.

The study was approved by the Mayo Clinic Institutional Review Board (#23-010273) and is currently open to accrual (ClinicalTrials.gov ID: NCT06386315) at all sites of Mayo Clinic (MN, AZ and FL).

## Stratification, Randomization, and Protocol Treatment

Stratification for this study will be based on disease stage (I/II versus III/IV/relapse/refractory) and enrollment site.

Patients will then be randomly assigned to 1 of 2 arms. In arm 1 (experimental arm), the prescription dose will be 9 Gy delivered in 3 fractions of 3 Gy per fraction, or 8 Gy delivered in 2 fractions of 4 Gy per fraction. In certain cases, as determined by the treating physician, 10 Gy delivered in 5 fractions may be used, such as for stomach, orbit, or other sites, at the physician’s discretion. In arm 2 (standard of care arm), the prescription dose will be 24 Gy delivered in 12 fractions of 2 Gy per fraction.

RT target delineation will follow the International Lymphoma Radiation Oncology Group guidelines for involved-site RT for nodal^8^ and extranodal^9^ NHL and National Clinical Trials Network (NCTN)^10^ guidelines.^11^

## Outcomes Measures

Acute and late toxicity (CTCAE version 5.0) will be assessed at baseline, end of treatment, day 7 post-RT, day 14 post-RT, and at 3 months.

Patient-reported outcomes (FACIT-Fatigue scale) will be collected at baseline, end of treatment, day 7 post-RT, and 3 months post-RT completion.

Financial toxicity, as determined by the COST-FACIT QOL, will be assessed at the end of treatment, with patients completing a questionnaire at the end of their radiation treatment.

Financial health care expenditure will be determined based on the base cost rates from Mayo Clinic’s reimbursement database for all codes related to the planning, treatment, and management.

Response rate will be obtained during follow-up imaging and clinical exam at 3 months post radiation treatment. Local recurrence rate will be obtained during follow-up imaging and clinical exam at 3 months, 6 months, 12 months, 18 months, and 24 months post radiation treatment. Relapse-free and overall survivals will be assessed during follow-up at 3 months, 6 months, 12 months, 18 months, and 24 months post radiation treatment.

## Statistical Consideration

### Decision rule

A difference of proportions of acute adverse events within experimental arm versus standard of care arm will be evaluated using the 2-group χ^2^ test of equal proportions. If the *P* value of the statistical test is < .10, then there is a statistically significant difference between the experimental regimen and standard of care arm in the direction of the observed proportional difference. Therefore, a significant difference in the direction of the experimental arm showing better outcomes, or reduced toxicity, will be worth consideration in future trials. It is also important to consider the local control rate when deciding whether to pursue further study. If the observed 2-year probability of local control is more than 7% inferior with 8 to 10 Gy versus 24 Gy, then we will not consider future trials.

### Sample size and study duration

The study is designed as a 2-arm randomized phase 2 trial with a total evaluable sample size of 102 patients and a 1:1 randomization (51 patients per arm). The overall study duration is expected to be 48 months.

### Power and significance levels

A 2-group χ2 test of equal proportions with a 2-sided α = 0.10 will be used to test the primary hypothesis based on acute toxicity estimates observed in the FoRT trial (grade 2+ acute toxicity of approximately 31% in the 24-Gy arm and approximately 9% in the 4-Gy arm). We hypothesize that the acute toxicity rate in the 24-Gy arm will remain similar, whereas we anticipate an acute toxicity rate of 10% in the 8- to 10-Gy arm. With a 2-sided alpha of 0.10, we will achieve 80% power to detect a difference in proportions of 0.21, assuming acute toxicity rates of 0.31 in the standard of care arm and 0.10 in the experimental arm. To ensure the evaluability of our primary endpoint, we will accrue 10 additional patients (56 per arm) to account for approximately 10% lost to follow-up.

### Primary analysis

The primary objective is to compare acute toxicity rates within the first 2 weeks after radiation between treatment arms using a χ2 test. Acute toxicity, defined as grade ≥ 2 adverse events at least possibly related to radiation treatment (per CTCAE v5.0), will be analyzed in all patients who started treatment. A 2-sided *P* value < .10 will indicate statistical significance. Absolute risk difference and relative risk will be calculated, with 95% confidence intervals reported using the score method.

### Secondary analyses

Secondary analyses will assess response rate, local control, and quality of life. Response rates between treatment arms will be compared using a χ2 test, with absolute risk difference, relative risk, and 95% confidence intervals reported. Local control rates at 3, 6, 12, 18, and 24 months will be estimated using the Kaplan–Meier method, with hazard ratios and 95% confidence intervals calculated via Cox proportional hazards models. Quality of life will be evaluated through FACIT-Fatigue scores collected from baseline to 3 months postradiation, with the fatigue subscale score (ranging from 0-52, where higher scores indicate better quality of life) analyzed using a longitudinal mixed-effects regression model. Differences in score trajectories between treatment arms will be assessed using likelihood ratio tests.

### Exploratory analyses

Exploratory analyses will include financial toxicity, health care expenditure, and correlative biomarker assessments. Financial toxicity will be evaluated at the end of treatment using a modified COST-FACIT questionnaire. Health care expenditure will be assessed using a linear regression model to compare costs between treatment arms. Additionally, correlative biomarker analysis will involve multivariable models to evaluate associations between biomarkers and both local control and treatment response, adjusting for potential confounders.

## Conclusion

This study aims to personalize RT for indolent NHL and determine whether intermediate reduced-dose hypofractionated RT can achieve comparable disease control while minimizing toxicity and treatment burden.

## Appendix Supplementary materials

MC230808_Protocol_MCCCCAmend#2_clean_17Jan2025 Image, application 1
