# Supplementary material for: Reduced-Dose Hypofractionated Radiation Therapy (3 Gy × 3 Fractions) for Indolent Non-Hodgkin’s lymphoma (POSEIDON): A Multisite Phase 2 Randomized Trial Protocol
Source: Adv Radiat Oncol. 2025 Sep 26;11(1):101908. doi: 10.1016/j.adro.2025.101908 (PMC12686686; doi:10.1016/j.adro.2025.101908)

Mayo Clinic Cancer Center

# MC230808: Reduced dose hypofractionated radiotherapy (3 Gy x 3 fractions) for indolent non-Hodgkin lymphoma (POSEIDON): a multisite phase 2 randomized trial

Sponsor/Principal Investigator: Bradford S. Hoppe, MD

Mayo Clinic Florida

Department of Radiation Oncology

4500 San Pablo Rd

Jacksonville, FL 32224

904-953-4203

Co-Principal Investigators: Scott C. Lester, MD; Mayo Clinic Rochester

William G. Rule, MD; Mayo Clinic Arizona

James Leenstra, MD; Mayo Clinic Health System – Northfield, MN

John Yeakel, MD; Mayo Clinic Health System – Albert Lea, MN

Now Bhar Alam, MD; Mayo Clinic Health System – Eau Claire, WI

Abigail Stockham, MD; Mayo Clinic Health System – LaCrosse, WI

Ron Smith, MD; Mayo Clinic Health System – Mankato, MN

Co-Investigators: Omran Saifi, MD; Mayo Clinic Florida (Radiation Oncology)

Jennifer L. Peterson, MD; Mayo Clinic Florida (Radiation Oncology)

Jason R. Young, MD; Mayo Clinic Florida (Radiology)

Manoj K. Jain, MD; Mayo Clinic Florida (Radiology)

Liuyan (Jennifer) Jiang, MD; Mayo Clinic Florida (Pathology)

Han W. Tun, MD; Mayo Clinic Florida (Medical Oncology)

Statistician: Lauren E. Haydu, PhD.√

Study Contributor: Anna C. Harrell, MPH, CCRP√

√ Study contributors not responsible for patient care

**Trial Supported by:**

**Funding:** MCCCC Lymphoma DG

| **Document History** | **Effective Date** |
| --- | --- |
| Activation | 04/30/2024 |
| Version 1.0  Version 2.0 | 0906/2024  *pending* |
|  |  |

# Protocol Resources

Refer to Protocol Catalog for study personnel

| **Questions:** | **Contact Name** |
| --- | --- |
| Patient eligibility*, test schedule, treatment delays/interruptions/adjustments, dose modifications, adverse events, forms completion and submission, protocol document, consent form, regulatory issues | Cancer Center Clinical Trials (contact for all Mayo locations)  Phone: (507) 293-6386 |
| Serious Adverse Event Reporting | FDA Coordination Team  Email: [INDSafety@mayo.edu](mailto:INDSafety@mayo.edu) |

# Table of Contents

[MC230808 Reduced dose hypofractionated radiotherapy (3 Gy x 3 fractions) for indolent non-Hodgkin lymphoma (POSEIDON): a multisite phase 2 randomized trial 1](#_Toc153886000)

[Protocol Resources 2](#_Toc153886001)

[Table of Contents 3](#_Toc153886002)

[Schema 4](#_Toc153886003)

[List of Abbreviations 5](#_Toc153886004)

[**1.0** **Background** 6](#_Toc153886005)

[**2.0** **Goals** 7](#_Toc153886006)

[**3.0** **Eligibility** 8](#_Toc153886007)

[**4.0** **Test Schedule** 10](#_Toc153886008)

[**5.0** **Stratification Factors OR Grouping Factors** 12](#_Toc153886009)

[**6.0** **Registration/Randomization Procedures** 12](#_Toc153886010)

[**7.0** **Protocol Treatment** 14](#_Toc153886011)

[**8.0** **Dosage Modification Based on Adverse Events** 17](#_Toc153886012)

[**9.0** **Ancillary Treatment/Supportive Care** 18](#_Toc153886013)

[**10.0** **Safety Data Collection and Reporting** 18](#_Toc153886014)

[**11.0** **Treatment Evaluation/Measurement of Effect** 31](#_Toc153886015)

[**12.0** **Descriptive Factors** 32](#_Toc153886016)

[**13.0** **Treatment/Follow-up Decision at Evaluation of Patient** 32](#_Toc153886017)

[**14.0** **Body Fluid Biospecimens** 33](#_Toc153886018)

[**15.0** **Drug/Device Information** 33](#_Toc153886019)

[**16.0** **Statistical Considerations and Methodology** 33](#_Toc153886020)

[**17.0** **Pathology Considerations/Tissue Biospecimens** 41](#_Toc153886021)

[**18.0** **Records and Data Collection Procedures** 42](#_Toc153886022)

[**19.0** **Budget** 43](#_Toc153886023)

[**20.0** **References** 43](#_Toc153886024)

[Appendix I: ECOG Performance Status 45](#_Toc153886025)

[Appendix II: Patient Questionnaires 46](#_Toc153886026)

[Appendix III: Criteria for Response Assessment 50](#_Toc153886027)

# Schema

**9 Gy in 3 fractions (experimental arm)**

**24 Gy in 12 fractions (SOC control arm)**

**Observation and Follow-up per protocol**

**Prescreen**

**Confirm eligibility through pathology and histology results and inclusion/exclusion criteria**

**1:1 Randomization**

**10 Gy in 5 fractions or 8 Gy in 2 fractions are acceptable alternatives at the physician discretion**

Duration of Participation: 24 months

Sample size: 102 patients

Subjects will be enrolled and followed on this study until 24 months after the completion of radiation treatment.

**List of Abbreviations**

| AE | Adverse Event/Adverse Experience |
| --- | --- |
| ISRT | Involved – site radiation therapy |
| CRF | Case Report Form |
| DSMB | Data and Safety Monitoring Board |
| SOC | Standard of Care |
| NHL | Non-Hodgkin’s Lymphoma |
| RT | Radiation Therapy |
| PI | Principal Investigator |
| IRB | Institutional Review Board |
| BED | Biological Effective Dose |
| OS | Overall survival |
| LC | Local control |
| ECOG | Eastern Cooperative Oncology Group |
| CTV | Clinical Tumor Volume |
| OTV | Optimization Target Volume |
| CT | Computed Tomography |
| PET | Positron Emission Tomography |
| QoL | Quality of Life |
| SOC | System Organ Class |
| MCCC | Mayo Clinic Cancer Center |
| IHC | Immunohistochemistry |
| FISH | Fluorescence in situ hybridization |

## **1.0 Background**

### Indolent Non-Hodgkin’s Lymphoma (NHL) constitutes around 40% of all NHL cases, with follicular lymphoma being the most common subtype [1]. Several studies have described the outcomes for patients with early-stage follicular lymphoma treated with RT alone, demonstrating 10-year progression-free survival (PFS) rates of approximately 50% and overall survival (OS) rates of 60% to 70% [2-4]. Various radiation techniques and doses were used during the different time periods covered in these studies. For example, patients received involved field radiation therapy (IFRT), extended-field radiation therapy (EFRT), total lymphoid irradiation (TLI), or sub-TLI (STLI) to doses of 35 Gy to 50 Gy at 1.5 Gy to 2.5 Gy per fraction. Relapses following radiation alone usually occurred at unirradiated sites [3]. Furthermore, a subsequent large study utilizing the Surveillance Epidemiology and End Results (SEER) registry found that including radiation in the treatment is associated with superior disease-specific and overall survival [5].

### Studies evaluating the use of RT in patients with early-stage indolent lymphoma have typically used radiation doses between 20 Gy–50 Gy. Although retrospective studies have demonstrated that lower radiation doses appeared to be as effective as higher doses, a recent phase III randomized study from the United Kingdom tested the results of 24 Gy versus 40 Gy–45 Gy, and showed similar overall response rate, local control rate, PFS and OS [6]. Thus, lower doses of 24 Gy to 30 Gy are generally preferred due to the anticipated lower risk of radiation-induced acute and late toxicity. Owing to improvements in staging, modern technology, use of image guidance and concern for radiation toxicity, treatment fields have also decreased from TLI, to extended field radiation (EFRT), to IFRT, and most recently to involved site radiation (ISRT) / involved node radiation (INRT) as it was shown to be equally effective and less toxic [7].

### Given the extreme radiosensitivity of indolent lymphoma and efficacy of lower doses of radiation, retrospective studies showed that 4 Gy in 2 fractions, specifically for orbital indolent lymphoma, achieved high response rate with minimal toxic effects, much shorter treatment course and lower cost [8]. Unfortunately, prospective attempts to replicate this reduction of RT dose for follicular and marginal zone NHL showed inferior disease control [9]. The FoRT study randomized patients with indolent lymphoma at 2 Gy/fraction to either 24 Gy or 4 Gy total radiation dose, which translates to a biologically effective dose (BED) of 29 Gy and 5 Gy when using an alpha/beta ratio of 10. The 2-year local progression-free rate was 94.1% after 24 Gy and 79.8% after 4 Gy demonstrating superiority in the 24 Gy arm. However, toxicity rates were significantly worse with 24 Gy compared to 4 Gy (Grade 2+ acute toxicity: 34% vs. 10%) [9]. While 24 Gy remains the standard of care based on disease control, many radiation oncologists continue to use 4 Gy as initially treatment and will use 20-24 Gy for those patients who develop a local relapse after 4 Gy without compromising overall survival. .

### This leaves us with important unanswered questions. 1) Can we find a more optimal intermediate dose between 4 Gy and 24 Gy that is similarly effective with respect to disease control as 24 Gy but has shorter treatment course, lower clinical and financial toxicity, and less financial health care expenditure? 2) Can we personalize RT for patients with indolent NHL by identifying prognostic predictors of local control and radio-resistance?

### Currently, risk stratification for indolent lymphoma includes the FLIPI and FLIPI 2, which use traditional prognostic factors based on blood tests (LDH, beta microglobulin) and CT imaging for size and sites involved, but do not take into consideration more contemporary techniques, such as functional imaging segmentation values and molecular classification. Recent studies revealed that functional imaging 18-fluoro-deoxyglucose (FDG) metabolic tumor volume (MTV) and total lesions glycolysis (TLG) serve as prognostic factors in indolent lymphoma. High MTV and TLG were associated with shorter time to treatment initiation and worse outcome [10-12]. These studies, however, focused on outcomes following systemic therapy and we do not have data on impact of functional imaging for patients with indolent lymphoma undergoing radiotherapy. Furthermore, studies have shown worse survival in patients with indolent lymphoma who have increased expression of TP53, MYC, BCL2, and FOXP1 [13-15].

### To date, we lack the optimal intermediate RT dose and the multidisciplinary approach that utilizes the clinical, molecular, and radiological features for indolent lymphoma to personalize radiotherapy treatment.

### Therefore, the purpose of this project is to evaluate reduction of radiotherapy dose for patients with indolent NHL by conducting a prospective randomized phase 2 study comparing reduced dose hypofractionated ISRT of 9 Gy delivered over 3 fractions (BED 12 Gy) to standard of care 24 Gy delivered over 12 fractions (BED 29 Gy), while simultaneously evaluating prognostic value of pre-radiation functional imaging parameters and molecular markers to predict treatment response and failure.

## **2.0 Goals**

### 2.1 Primary Goal

The planned study is a randomized Phase 2 trial with the primary goal to show that the experimental arm (9 Gy in 3 fractions, 8 Gy in 2 fractions, or 10 Gy in 5 fractions) has significantly reduced acute toxicity (grade ≥ 2 adverse events at least possibly related to radiation treatment within 14 days after the end of radiation treatment (according to CTCAE v5.0)) compared to 24 Gy in 12 fractions.

### 2.2 Secondary Goals

The secondary objectives of the study are to evaluate:

- Patient Reported Quality of life [timeframe: 7 days after radiation treatment end date]
- Response rate [timeframe: at 3 months (+/- 14 days) post radiation treatment start date]
- Local control rate [timeframe: up to 24 months after the completion of radiation treatment]
- Relapse-free survival [timeframe: up to 24 months after completion of radiation treatment]

2.3 Exploratory Goals

- Financial toxicity will be assessed at the end of radiation treatment.
- Financial Health Care Expenditure will be assessed at the end of radiation treatment
- Late Toxicity. Late adverse events will be assessed at 3 months post radiation treatment. Toxicity refers to those adverse events that are possibly related to radiation treatment.

2.4 Correlative Research

2.4.1 Biopsies of enrolled patients will be evaluated for pathological assessment of cellular and genetic mutations to correlate them with disease local relapse and radiation resistance. This includes:

- TP53 gene alteration by Fluorescence in situ hybridization (FISH)
- Expression of TP53, MYC and proliferation index (Ki-67) by immunohistochemistry stain (IHC)

#### 2.4.2 Patients will have their baseline PET/CT scan undergo auto-segmentation to calculate the functional imaging 18-fluoro-deoxyglucose (FDG) metabolic tumor volume (MTV), total lesions glycolysis (TLG) and maximum standardized uptake volume (SUVmax) of the sites to be treated with ISRT using MIMvista platform to correlate it with disease local relapse and treatment response.

## **3.0 Eligibility**

### 3.1 Registration – Inclusion Criteria

#### 3.1.1 Age ≥18 years

#### 3.1.2 Histological confirmation of indolent B-cell lymphoma that can include any of the following:

#### Follicular lymphoma (grade 1 or 2 or 3A)

#### Marginal zone lymphoma (Nodal or extranodal)

Follicle center lymphoma

#### 3.1.3 Any stage disease

#### 3.1.4 Initial, refractory, or relapsed disease. If relapse involves the site to be treated there must be evidence of disease progression

#### 3.1.5 ECOG Performance Status (PS) ≤3 (Appendix I)

#### 3.1.6 Negative pregnancy test done ≤7 days prior to registration, for persons of childbearing potential only

#### 3.1.7 Provide written informed consent

#### 3.1.8 Ability to complete questionnaire(s) by themselves

#### 3.1.9 Willing to return to enrolling institution for follow-up (during the Active Monitoring Phase of the study). Virtual visits can also be considered as an option for applicable items

3.1.10 Confirmation from radiation oncologist of suitability to participate in study

### 3.2 Registration – Exclusion Criteria

#### 3.2.1 Any of the following:

#### Pregnant women

#### Nursing women

#### Men or women of childbearing potential who are unwilling to employ adequate contraception

#### 3.2.2 T-cell lymphoma

#### 3.2.3 Small and chronic lymphocytic lymphoma

#### 3.2.4 Grade 3B follicular lymphoma

##

## **4.0 Test Schedule**

|  |  | **Active Monitoring Phase** | | | **Clinical Follow-Up** | | |
| --- | --- | --- | --- | --- | --- | --- | --- |
| **Tests and procedures** | ***≤ 45 days prior to registration*** | ***End of Treatment*** | ***Day 7***  ***post-ISRT*** | ***Day 14***  ***post-ISRT*** | ***3 Months***  ***post-RT^1^*** | ***6 Months***  ***post-RT*** | ***Every clinically indicated follow-up***  ***(q 6 months)^1^*** |
| Window |  | ±1 day | ±2 days | ±2 days | ±14 days | ±30 days | ±30 days |
| History and exam, PS^2^ | X |  |  |  | X | X | X |
| Radiation Oncology consult^2^ | X |  |  |  |  |  |  |
| Adverse Event assessment^3^ | X | X | X | X | X |  |  |
| Pregnancy test^2,^4 | X |  |  |  |  |  |  |
| Hematology group: CBC with 5-part differential  (HgB, WBC, PLT, LDH)^2^ | X |  |  |  |  |  |  |
| PET/CT ^2^ | X  (90 days) |  |  |  | X  (strongly consider, unless FDG negative at baseline) | X* | X* |
| CT Chest/Abdomen/Pelvis +/- Neck^2, 7^ |  |  |  |  | X  (if PET not done) | X* | X* |
| Endoscopy with gastric biopsy (only if gastric lymphoma)^2^ | X  (***≤*** 90 days) |  |  |  | X^6^ | X** |  |
| Patient Reported Outcomes (Appendix II: FACIT-Fatigue)^3^ | X | X | X |  | X |  |  |
| Financial Toxicity Questionnaire  (Appendix II: COST-FACIT)^3^ |  | X |  |  |  |  |  |
| Optional Research,  tissue specimen5^,R^ |  | X |  |  |  |  |  |

Radiation treatment (RT) = 3 vs. 12 fractions (5 days vs. 16 days ±2 days)

^1^ Follow-up assessments will be completed as per clinician’s discretion.

^2^ Standard of care

^3^ Research non-billable

^4^ For persons of childbearing potential only. Must be done ≤7 days prior to registration.

^5^ Historic tissue specimen may be pulled and used for analysis by end of study.

^6^ +/- 30 days

^7^ CT and/or PET should not be mandated for patients with primary cutaneous lymphoma where physical exam is used for evaluation of treatment response..

^R^ Research funded

* CT chest/abdomen/pelvis +/- neck or PET/CT or PET/MRI ok at these time points

** If 3 month biopsy was positive

Event monitoring is the period when the participant is no longer following the protocol test schedule due to disease progression and/or the patient was started on a new non-study anti-cancer treatment. During Event Monitoring, the data collection schedule is dictated by the protocol, but the visit schedule is determined by clinical practice at each participating site. During the Event Monitoring Phase of the study, the participant is being monitored for key study events such as new non-study anti-cancer treatments given, new primaries, and death. Samples from biospecimens collected in the course of clinical care may be requested but cannot be required of the participant.

##

## **5.0** **Stratification Factors OR Grouping Factors**

Stage I/II vs. Stage III/IV/relapse/refractory

Enrollment site (FL, AZ, MN)

## **6.0 Registration/Randomization Procedures**

### 6.1 Registration (Step 1)

#### 6.1.1 Registering a Patient

To register a patient, access the Mayo Clinic Research Registration Application at <https://registration.mayo.edu>. The registration/ randomization application is available 24 hours a day, 7 days a week. Back up and/or system support contact information is available on the website. If unable to access the website, email Research Registration Office at [random01@mayo.edu](mailto:random01@mayo.edu) between the hours of 8 a.m. and 4:30 p.m. Central Time (Monday through Friday).

The instructions for the registration/randomization application are available on the Mayo Clinic Office of Clinical Trials web page (<https://www.mayo.edu/research/centers-programs/center-clinical-translational-science/offices/office-of-clinical-trials/research-registration-application>) and detail the process for completing and confirming patient registration. Prior to initiation of protocol treatment, this process must be completed in its entirety and an MCCC subject ID number must be available as noted in the instructions. It is the responsibility of the individual registering the patient to confirm the process has been successfully completed prior to release of the study agent. Patient registration via the registration/randomization application can be confirmed in any of the following ways:

- Contact the Research Registration Office at [random01@mayo.edu](mailto:random01@mayo.edu). If the patient was fully registered, the Research Registration Office staff can access the information from the centralized database and confirm the registration.
- Refer to the Research Registration Application training course on the Mayo Clinic Office of Clinical Trials webpage (link above) for instructions on viewing the patient’s registration confirmation.

### 6.2 Randomization Procedures

#### 6.2.1 The factors defined in **Section 5.0** will be used as stratification factors.

#### 6.2.2 After the patient has been registered into the study, the values of the stratification factors will be recorded, and the patient will be assigned to one of the following treatment groups using the Pocock and Simon dynamic allocation procedure, which balances the marginal distributions of the stratification factors between the treatment groups (Pocock and Simon 1975).

- Arm 1: The prescription dose will be 9 Gy delivered in 3 fractions of 3 Gy per fraction or 8 Gy delivered in 2 fractions of 4 Gy per fraction . In some cases, the investigator can use 10 Gy delivered in 5 fractions (stomach/orbit/other at treating MD’s discretion).
- Arm 2: The prescription dose will be 24 Gy delivered in 12 fractions of 2 Gy per fraction.

### 6.3 Verification of Materials

Prior to accepting the registration, registration/randomization application will verify the following:

- IRB approval at the registering institution
- Patient eligibility
- Existence of a signed consent form
- Existence of a signed authorization for use and disclosure of protected health information

### 6.4 Documentation of IRB Approval

Documentation of IRB approval must be on file in the Registration Office before an investigator may register any patients.

In addition to submitting initial IRB approval documents, ongoing IRB approval documentation must be on file (no less than annually) with the Research Registration Office (fax: 507-284-0885 or email: [random01@mayo.edu](mailto:random01@mayo.edu)). If the necessary documentation is not submitted in advance of attempting patient registration, the registration will not be accepted, and the patient may not be enrolled in the protocol until the situation is resolved.

When the study has been permanently closed to patient enrollment, submission of annual IRB approvals to the Registration Office is no longer necessary.

### 6.5 Correlative Research

#### 6.5.1 Optional

An optional correlative research component is part of this study, there will be an option to select if the patient is to be registered onto this component (see
**Section 17.0**).

###

### 6.6 Treatment on Protocol

Treatment on this protocol must commence at approved Mayo Clinic sites under the supervision of the trained medical team.

### 6.7 Treatment Start

Treatment cannot begin prior to registration and must begin ≤ 21 days after registration.

### 6.8 Pretreatment

Pretreatment tests/procedures (see **Section 4.0**) must be completed within the guidelines specified on the test schedule.

### 6.9 Baseline Symptoms

All required baseline symptoms (see **Section 10.2.1.2**) must be documented and graded.

### 6.10 Radiation Oncology Consult Required

A radiation oncologist has seen the patient and confirms the patient is a suitable candidate for this study.

### 6.11 Patient Questionnaire Booklets

Patient questionnaire booklets can be found in the Protocol Catalog and printed for each patient.

### 6.12 Study Conduct

The clinical trial will be conducted in compliance with regulations (21 CFR 312, 50, and 56), guidelines for Good Clinical Practice (ICH Guidance E6), and in accordance with general ethical principles outlined in the Declaration of Helsinki; informed consent will be obtained from all participating patients; the protocol and any amendments will be subject to approval by the designated IRB prior to implementation, in accordance with 21 CFR 56.103(a); and subject records will be stored in a secure location and subject confidentiality will be maintained. The investigator will be thoroughly familiar with the appropriate use of the study intervention as described in the protocol and Investigator’s Brochure. Essential clinical documents will be maintained to demonstrate the validity of the study and the integrity of the data collected. Master files should be established at the beginning of the study, maintained for the duration of the study and retained according to the appropriate regulations.

####

## **7.0 Protocol Treatment**

### 7.1 Radiation Therapy

7.1.1 Target delineation

Target delineation should follow the International Lymphoma Radiation Oncology Group (ILROG) guidelines for involved site radiation therapy (ISRT) for nodal and extranodal NHL and will be at the discretion of the treating physician.

Per ISRT guidelines, a Clinical Target Volume (CTV) and a Planned Target Volume (PTV) should be included. In situations where the target is moving, then an ITV should be used in place of the CTV.

7.1.2 Treatment planning and delivery

Treatment can be delivered using photons, protons, or electrons at the treating physician’s discretion. When treating with photons; 3D-conformal radiotherapy (3D-CRT), intensity modulated radiotherapy (IMRT), or volumetric arc radiotherapy (VMAT) are all allowed.

Arm 1: The prescription dose will be 9 Gy delivered in 3 fractions of 3 Gy per fraction or 8 Gy delivered in 2 fractions of 4 Gy per fraction. Treatment will be delivered one fraction a day except on weekends and holidays. Note that at the treating physician’s discretion (such as for gastric, dural, orbital, other lymphoma), 10 Gy in 5 fractions is allowed as an alternative ifractionation.

Arm 2: The prescription dose will be 24 Gy delivered in 12 fractions of 2 Gy per fraction. Treatment will be delivered one fraction a day except on weekends and holidays.

7.1.3 Target coverage and normal tissue constraints are outlined below (if needed)

|  | **Constraints Goals** | | | **Highest Priority Constraints** | | |
| --- | --- | --- | --- | --- | --- | --- |
| **Structure** | **DVH Endpoint** | **Constraint** | **Priority** | **DVH Endpoint** | **Constraint** | **Priority** |
| CTV or ITV | D99%[%] | >=100% | 2 | D95%[%] | >=100% | 1 |
|  | V110% |  | Report | - | - | - |
| PTV | D95%[%] | >=99% | 2 | D95%[%] | >=95% | 1 |
|  | V110% |  | Report | - | - | - |
| Ipsilateral Parotid | Mean | <11 Gy | 2 | Mean | <24 Gy | 1 |
| Ipsilateral submandibular gland | Mean | <11 Gy | 2 | Mean | <24 Gy | 1 |
| Oral Cavity | Mean | <11 Gy  <6 Gy @ 3 Gy/fxn | 2 | Mean | <24 Gy | 1 |
| Ipsilateral Lacrimal gland | Mean | <11 Gy | 2 | Mean | <24 Gy | 1 |
| Larynx |  |  |  | Mean | <25 Gy | 1 |
| Constrictors_pharyng |  |  |  | Mean | <25 Gy |  |
| Heart | Mean | <8 Gy  <4 Gy @ 3 Gy/fxn | 2 | Mean | <15 Gy  <6 Gy @ 3 Gy/fxn | 1 |
| Left ventricle | Mean | <8 Gy  <4 Gy @ 3 Gy/fxn | 2 | Mean | <15 Gy  <6 Gy @ 3 Gy/fxn | 1 |
| Lung | Mean | <10 Gy  <4 Gy @ 3 Gy/fxn | 2 | Mean | <13.5 Gy  <6 Gy @ 3 Gy/fxn | 1 |
|  | V20 | <20% | 2 | V20 | <30% | 1 |
|  |  |  |  | V5 | <55% | 1 |
| Liver | Mean | <15 Gy |  | Mean | <20 Gy | 1 |
|  |  |  |  | V20 | <30% | 1 |
| Stomach | Mean | <25 Gy | 2 | Mean | <30 Gy | 1 |
| Spleen | Mean | <10 Gy | 2 |  |  |  |
| Pancreas | Mean | <10 Gy | 2 |  |  |  |
| Small Bowel | V15 Gy | <120cc | 2 |  |  |  |
| Kidney_total | Mean | <8 Gy  <3 Gy @ 3 Gy/fxn | 2 | Mean | <12 Gy  <5 Gy @ 3 Gy/fxn | 1 |
| Kidney_total | V20 | <15% | 2 | V20 | <25% | 1 |

7.2 Follow up Visits

7.2.1 Acute Toxicity follow-up

The clinical team will follow up with the patient in person, virtually or over the phone every week during treatment and following treatment weekly for an additional 2-weeks post radiation treatment to assess the acute toxicity profile via flowsheets in Epic.

7.2.2 Follow-up imaging

Standard of care PET or CT scans will be used for follow-up imaging +/- endoscopy for gastric lymphoma. These follow-up imaging +/- procedures will occur at 3 months, 6 months, 12 months, 18 months, and 24 months after the completion of radiation treatment per the test schedule and associated windows described in **Section 4.0**. Additional endoscopies after 3 months are at the treating physician’s discretion. For patients without measurable disease by CT or PET/CT scan at baseline (eg cutaneous lymphoma), follow up imaging is not required.

7.2.3 Outcomes

- Adverse events (CTCAE version 5.0) will be assessed at Baseline, End of Treatment, Day 7 post-RT, Day 14 post-RT, and at 3 months within the windows defined in **Section 4.0**. The primary outcome is acute toxicity, defined as any adverse event grade 2 or higher at least possibly related to radiation treatment that occurs up to 14 days after the end of radiation treatment.
- Patient reported outcomes (FACIT-Fatigue scale) will be collected at Baseline, End of Treatment, Day 7 post-RT and 3 months post-RT within the windows defined in **Section 4.0**.
- Financial toxicity, determined by the COST-FACIT QOL, will be evaluated after RT at the end of treatment (+/- 1 day).
- Financial health care expenditure (see **Section 7.2.8**) will be collected after RT at the end of treatment.
- Response rate will be obtained during follow-up imaging and clinical exam at 3 months (+/- 14 days) post radiation treatment.
- Local recurrence rate will be obtained during follow-up imaging and clinical exam at 3 months, 6 months, 12 months, 18 months, and 24 months post radiation treatment within the windows defined in **Section 4.0**.
- Overall survival will be assessed during follow-up at 3 months, 6 months, 12 months, 18 months, and 24 months post radiation treatment per clinician's discretion within the windows defined in **Section 4.0**.

7.2.4 Acute and late adverse event monitoring

Adverse events will be obtained as detailed above as per CTCAE v5.

7.2.5 Quality of Life

Patient Reported Outcomes will be collected at Baseline, End of Treatment, Day 7 post-RT, and 3 months post-RT completion date and will be based on the FACIT-Fatigue questionnaire within the windows defined in **Section 4.0**.

7.2.6 Financial toxicity

Financial toxicity will be determined using a questionnaire completed by the patient at the end of their ISRT (fraction 3 for experimental arm and fraction 12 for the SOC arm). This questionnaire will entail the validated FACIT-COST scale with added questions to assess specifically for travel and accommodation expenses, travel distance and time, and loss of work time for patient and care givers.

7.2.7 Financial Health Care Expenditure

Financial health care expenditures will be determined by first obtaining base cost rates from Mayo Clinic’s reimbursement database for all codes included in the planning, treatment, and management of patient’s enrolled in both the 3 and 12 fractions arms. Mean total radiotherapy costs will then be calculated using inflation adjusted standardized Medicare rates and directly compared between the 2 treatment arms.

7.2.8 Dose Modification Based on Adverse Events

No dose modifications planned, aside from early stopping rules (below).

## **8.0 Dosage Modification Based on Adverse Events**

Aside from early stopping rules (below), dose modification might be adopted if the hypofractionation regimen was associated with increased adverse events in the first 10 accrued patients. Specifically, the experimental arm will be monitored, after 10 patients are treated per arm, for less than 40% of patients experiencing a grade 3+ adverse event possibly related to treatment within 90 days post-treatment. If that threshold is crossed, the study will temporarily be suspended, and the experimental arm will be adapted to 10 Gy in 5 fractions.

## **9.0 Ancillary Treatment/Supportive Care**

### 9.1 Full supportive care

Patients should receive full supportive care while on this study. This includes blood product support, antibiotic treatment, and treatment of other newly diagnosed or concurrent medical conditions. All blood products and concomitant medications such as antidiarrheals, analgesics, and/or antiemetics received from the first day of study treatment administration until 30 days after the final dose will be recorded in the medical records.

9.2 Steroids and other supportive medications (Antiemetics, antidiarrheals etc.)

Steroids and other supportive medications may be used at the discretion of the attending physician.

## **10.0 Safety Data Collection and Reporting**

### 10.1 Definitions

#### 10.1.1 Investigator

An investigator includes the Site Principal Investigator (PI) or a designated Sub-Investigator.

Sub-Investigator

A Sub-Investigator is a medically qualified delegate who has been identified and confirmed by the Site PI. Medically qualified designations may include, but are not limited to:

- Medical Doctorate (M.D.), or equivalent medical school graduate degree
- Physician Assistant (P.A.)
- Certified Nurse Practitioner (C.N.P.)
- Registered Nurse (R.N.)

#### 10.1.2 Adverse Event (AE)

An adverse event is defined as any untoward medical occurrence in a clinical trial subject. The event does not necessarily have a causal relationship with study treatment. (FDA, 21 CFR 312.32; ICH E2A and ICH E6)

The definition of AEs includes:

- Worsening of a pre-existing medical condition. Worsening indicates that the pre-existing medical condition or underlying disease (e.g., diabetes, migraine headaches, gout) has increased in severity, frequency, and/or duration more than would be expected and/or has an association with a significantly worse outcome than expected.

NOTE: A pre-existing condition that has not worsened more than anticipated (i.e., more than usual fluctuation of disease) during the study or involves an intervention such as elective cosmetic surgery or a medical procedure while on study, is not considered an AE.

- Unintended or unfavorable sign or symptom
- A disease temporally associated with participation in the protocol
- An intercurrent illness or injury that impairs the well-being of the subject

In general, abnormal laboratory values or diagnostic test results constitute AEs only if they induce clinical signs or symptoms or require treatment or further diagnostic tests.

Hospitalization for elective surgery or routine clinical procedures that are not the result of an AE (e.g., surgical insertion of central line) should not be recorded as an AE.

Disease progression should not be recorded as an AE, unless it is attributable to the study regimen by the site investigator.

The investigator is responsible for ensuring that any AEs observed by the investigator or reported by the subject are recorded in the subject’s medical record.

When AE terms encompass multiple other AE terms in their definition, those AEs within the definition will not be separately recorded, graded or attributed.

- - - - For example, “Flu-like Symptoms” in CTCAE v5.0 is defined as, *“A disorder characterized by a group of symptoms similar to those observed in patients with the flu. It includes fever, chills, body aches, malaise, loss of appetite and dry cough.”* Therefore, if a patient is determined to have grade 2 flu-like symptoms unrelated to study procedures by the treating provider, the patient’s fever, chills, body aches, malaise, etc. will not be separately recorded, graded nor attributed.

#### 10.1.3 Serious Adverse Event (SAE)

A serious adverse event is defined as an adverse event that meets ***at least one*** of the following serious criteria (FDA, 21 CFR 312.32; ICH E2A and ICH E6):

- Fatal
- Life threatening (places the subject at immediate risk of death)
- Requires inpatient hospitalization for ≥24 hours or prolongation of existing hospitalization.

NOTE: Hospitalization for anticipated or protocol specified procedures such as administration of chemotherapy, central line insertion, metastasis interventional therapy, resection of primary tumor, or elective surgery, will not be considered SAEs.

- Results in persistent or significant disability/incapacity, congenital anomaly/birth defect.
- Is an important medical event (defined as a medical event(s) that may not be immediately life-threatening or result in death or hospitalization but, based upon appropriate medical and scientific judgment, may jeopardize the subject or may require intervention (e.g., medical, surgical) to prevent one of the other serious outcomes listed in the definition above)

NOTE: Examples of such events include, but are not limited to, intensive treatment in an emergency room or at home for allergic bronchospasm; blood dyscrasias or convulsions not resulting in hospitalization; or the development of drug dependency or drug abuse. (See **Table 10.2.2.1**)

If an investigator considers an event to be clinically important, but it does not meet any of the serious criteria, the event could be classified as a SAE under the criterion of “other medically important serious event.”

#### 10.1.4 Suspected Adverse Reaction

Any AE for which there is a reasonable possibility that the treatment caused the AE.

#### 10.1.5 Adverse Events of Special Interest (AESI)

Events that would not typically be considered to meet the criteria for expedited reporting, but that for a specific protocol are being reported via expedited means in order to facilitate the review of safety data (may be requested by the FDA or the sponsor).

#### 10.1.6 Protocol Violation/Deviation

Any change, divergence or departure from the study design or research procedures that has not been approved by the IRB.

**Major Protocol Violation/Deviation:** Any change that affects the rights and welfare of subjects and others, increases risks to subjects and others, decreases potential benefits, compromises the integrity or validity of the research, or represents willful or knowing misconduct.

**Minor Protocol Violation/Deviation:** Any change that did not increase the risk or decrease the benefit or significantly affect the subject’s rights, safety or welfare and/or the integrity of research data (e.g., a routine lab missed at a visit and re-drawn, shortening the duration between a planned study visit, using an outdated HIPAA form or consent form when there are no differences between the two forms other than the approval date).

#### 10.1.7 Medication Error

A medication error is defined as any accidental incorrect administration or dosing of a medicinal product.

#### 10.1.8 Expedited Reporting

Events immediately reported to sponsor using the appropriate iMedidata Rave SAE eCRF(s) once the study team has become aware of the event.

#### 10.1.9a Routine Reporting

Events reported to sponsor via the appropriate iMedidata Rave AE eCRF(s).

#### 10.1.9b Expected versus Unexpected Events

**Expected events** are those described within [**Section 15.0**](#_15.0_Drug/Device_Information) of the protocol, the study specific consent form, package insert (if applicable), and/or the investigator brochure (IB), (if an investigator brochure is not required, otherwise described in the general investigational plan).

**Unexpected adverse events** or suspected adverse reactions are those not listed in [**Section 15.0**](#_15.0_Drug/Device_Information) of the protocol, the study specific consent form, package insert (if applicable), or in the investigator brochure (or are not listed at the specificity or severity that has been observed); if an investigator brochure is not required or available, is not consistent with the risk information described in the general investigational plan.

An investigational agent/intervention might exacerbate the expected AEs associated with a commercial agent. Therefore, if an expected AE (for the commercial agent) occurs with a higher degree of severity or specificity, expedited reporting is required.

NOTE: Refer to protocol or IB for reporting needs.

#### 10.1.9c Relatedness

A problem or event is "related" if it is possibly related and/or attributed to the research procedures including (but not limited to) investigational product(s), assay process and/or study procedures or activity.

**The only study intervention for this clinical trial is radiation therapy and thus recorded AEs will only be attributed toward radiation therapy alone.**

**AEs ≤ Grade 1 will not be recorded on the AE assessment forms nor given an attribution due to purpose of the study and nature of patient’s disease progression.**

**AEs will no longer be followed or recorded on the AE assessment forms nor given an attribution if the patient starts a non-study, new anti-cancer treatment OR is moved into event monitoring due to disease progression.** Specifically, attributions will be categorized using the following terms:

| **Unrelated** | *AE is* ***not*** *related to study agent(s) or research procedure(s)* |
| --- | --- |
| **Unlikely** | *AE is* ***doubtfully*** *related to study agent(s) or research procedure(s)* |
| **Possible** | *AE* ***may be*** *related to study agent(s) or research procedure(s)* |
| **Probable** | *AE is* ***likely*** *related to study agent(s) or research procedure(s)* |
| **Definite** | *AE is* ***clearly*** *related to study agent(s) or research procedure(s)* |

#### 10.1.9d Severity Criteria

An assessment of the Adverse Events severity grade will be made by the investigator according to the NCI CTCAE Version 5.

A copy of this CTCAE version can be downloaded from the CTEP web site: (http://ctep.cancer.gov/protocolDevelopment/electronic_applications/ctc.htm)

### 10.2 Site Reporting

Subject data accrued on this study will be reported in accordance with 21CFR 312.32.

All AEs and SAEs will be reported using iMedidata Rave electronic data capture system. Please refer to the “Mayo Clinic Cancer Center Electronic Case Report Form (eCRF) Completion Guidelines” for additional instructions.

All AEs and SAEs must be evaluated, diagnosed, and assessed for safety reporting by an investigator.

The AE grading scale for this study will be Common Terminology Criteria for AEs (CTCAE) version 5. All appropriate treatment areas must have access to this CTCAE version.

#### 10.2.1 Adverse Events

10.2.1.1 Site Requirements for Reporting AEs

From the time of registration until 30 days after the administration of the last dose of study drug/agent or therapy, or until a new anti-cancer treatment starts (whichever occurs first), the Site Principal Investigator (PI) is responsible for ensuring that all AEs (observed by study team, provided by external sources, etc.) are reported to the sponsor if the meet the site reporting criteria outlined in Section 10.1.9c.

**After 30 days from the last day of radiation treatment, only adverse events that are attributed as possible, probable or definite to the radiation treatment and are** **≥ grade 2 are required to be recorded on the adverse event forms unless patient has received a non-study new anti-cancer treatment or been moved into event monitoring due to disease progression.** Also refer to **Section 10.2.2** for the reporting of any possible SAEs as applicable.

The investigator(s) is responsible for:

- - AE diagnosis or syndrome(s), if known (if not known, signs or symptoms)
  - Dates of onset and resolution (if resolved)
  - Severity
  - Assessment of the relationship to study treatment(s)
  - As applicable, identify and clarify if an adjustment in treatment dose occurred due to an AE
  - Determine the relationship of the AE with any study mandated activity (e.g., administration of investigational product, protocol-required therapies, and/or procedure (including any screening procedure(s))
  - Reviewing laboratory test results and determining whether an abnormal value in an individual study subject represents a clinically significant change from the subject’s baseline values
- In general, abnormal laboratory findings without clinical significance (based on the Investigator's judgment) are not to be recorded as AEs. Exceptions may include but are not limited to:
  - Baseline and Adverse Events Evaluations (**Section 10.2.1.2**)
- However, laboratory value changes that require treatment or adjustment in current therapy are considered AEs.
- Where applicable, clinical sequelae (not the laboratory abnormality) are to be recorded as the AE.
- Asymptomatic laboratory abnormalities that do not require treatment will not be collected as AEs.
  - Following reported AEs until resolution

The delegated study team member(s) must document, and report AEs as follows:

- - All AEs must be documented in the subject’s medical record and recorded on the appropriate study specific iMedidata Rave “Adverse Events” eCRF. Information documented on the iMedidata Rave “Adverse Events” eCRF must be consistent with that recorded on the source document.
- AEs will be recorded in the patient’s medical record regardless of whether or not they are considered related to the study intervention. However, only those AEs that are considered related to study intervention by site investigator and/or treating physician (Section 10.2.1.1) will be explicitly graded, attributed and followed until resolution to ≤Grade 1 or baseline, deemed clinically insignificant, patient is moved into event monitoring, and/or until a new anti-cancer treatment starts, whichever occurs first. Exceptions may include non-clinically significant labs if they meet non-critical reporting criteria per investigator discretion.
  - - If a subject is permanently withdrawn from protocol-required therapies because of an AE, the subject status must be submitted, via the appropriate iMedidata Rave eCRF.
    - If an AE results in adjustment in treatment dose (i.e., dose delay, dose reduction, etc.):
- The individual AE needs to be documented on iMedidata Rave “Adverse Events” eCRF and,
- The details of the dose adjustment due to the individual AE need to be documented on the iMedidata Rave “Treatment” (Intervention) eCRF.

10.2.1.2 Solicited Adverse Events Evaluations

Pre-treatment symptoms/conditions/adverse events must be graded at baseline and at each evaluation according to the test schedule in Section 4.0. Grading is per CTCAE v5.0 unless alternate grading is indicated in the table below:

| **CTCAE** **System Organ Class (SOC)** | **Adverse event/ Symptoms** | **CTCAE Grade at which the event will not be reported in an expedited manner^1^** |
| --- | --- | --- |
| General disorders and administrations site conditions | Fatigue | ≤Grade 3 |
|  | Pain | ≤Grade 3 |
|  | Dry eye | ≤Grade 3 |
| Skin and subcutaneous tissue disorders | Alopecia | ≤Grade 2 |
|  | Mucositis oral | ≤Grade 3 |
| Gastrointestinal disorders (GI) | Vomiting | ≤Grade 3 |
|  | Esophagitis | ≤Grade 3 |
|  | Dysphagia | ≤Grade 3 |
|  | Constipation | ≤Grade 3 |
|  | Nausea | ≤Grade 3 |
|  | Diarrhea | ≤Grade 3 |
|  | Dry mouth | ≤Grade 4 |
| Injury, poisoning and procedural complications | Dermatitis radiation | ≤Grade 3 |

#### 10.2.2 Serious Adverse Events

10.2.2.1 Site Requirements for Reporting SAEs

From the time of registration until ≤ 30 days after the administration of the last dose of study drug/agent or therapy, or until a new anti-cancer treatment starts (whichever occurs first), the Site Principal Investigator (PI) is responsible for ensuring that all SAEs (observed by study team, provided by external sources, etc.) are reported immediately to the sponsor, unless explicitly stated otherwise in table in Section 10.2.1.2

Any SAEs that occur when the patient is in event monitoring are not subject to being reported (expeditiously or otherwise) on this study unless they occur ≤ 30 days after the last dose of radiation the patient receives per protocol AND the patient has not started a new non-study anti-cancer treatment.

In addition to reporting to Mayo Clinic, the investigator is responsible for reporting to their appropriate IRB/IEC in accordance with local regulatory requirements and procedures.

Initial Reporting:

The investigator/study team must report SAEs as follows:

- - - All SAEs must be immediately submitted to Mayo Clinic following the study team’s knowledge of the event via the iMedidata Rave “Adverse Events” eCRF.

NOTES: This eCRF will generate the “SAE: Event Information” eCRF when the event is indicated as “serious.” Information documented on the iMedidata Rave “SAE: Event Information” eCRF must be consistent with that recorded on the source document.

Once the SAE information has been captured and submitted in iMedidata Rave, a report can be generated. A copy of this completed report must be kept within the study file at the study site.

- - - - The investigator must assess whether the SAE is possibly related to the investigational product.
      - The investigator is expected to follow reported SAEs until stabilization, resolution, or patient death. (See section below on Follow-Up Reporting.)

Follow-Up Reporting:

New information relating to a previously reported SAE needs to be submitted to Mayo Clinic:

- - - - Immediately upon site becoming aware of updated information
      - Sites may be required to:
- Provide additional information including but not limited to discharge summaries, medical records, etc.
- Query external locations for additional information if events occurred outside the participating site (i.e., Hospitalization records)
- Respond to Sponsor query(ies) if additional information is needed
  - - - All SAEs regardless of relation to study intervention will be followed until resolution to ≤ Grade 1 or baseline and/or deemed clinically insignificant, a new anti-cancer treatment starts, the patient is moved into event monitoring, or death whichever occurs first.
      - Revise the previously documented SAE on the appropriate iMedidata Rave eCRF.

NOTES: Information documented on the iMedidata Rave eCRF must be consistent with that recorded on the source document.

Once the updated SAE information has been captured and submitted in iMedidata Rave, a report can be generated. A copy of this updated report must be kept within the study file at the study site.

NOTE: Refer to **Section 10.2.3** for additional reporting requirements.

**Table 10.2.2.2 Expedited Reporting Requirements for Adverse Events/Serious Adverse Events**

| **EXPEDITED REPORTING REQUIREMENTS FOR SERIOUS ADVERSE EVENTS  (21 CFR Part 312)**  Investigators MUST immediately report to Mayo Clinic **ANY** Serious Adverse Events (SAE), whether or not they are considered related to the investigational agent(s)/intervention (21 CFR 312.64).  Mayo Clinic will report to FDA, per regulations [21 CFR 312.32(c)(d)].  The definition of a SAE can be found in **Section 10.1.3**.  Detailed site requirements for reporting SAEs can be found in **Section 10.2.2.1**. | | | |
| --- | --- | --- | --- |
| **Initial Reporting** | | **Follow-up Reporting** | |
| **HOSPITALIZATION**  **≥24 hours**  (Any Grade and/or Attribution) | **IMMEDIATELY** upon study team’s awareness of event.  (Not to exceed 24 hours.) | **HOSPITALIZATION**  **≥24 hours**  (Any Grade and/or Attribution) | **IMMEDIATELY** upon receiving any updated information regarding event.  (Not to exceed 24 hours.) |
| **GRADE 3**  Attribution of Possible, Probable, or Definite |  | **GRADE 3**  Attribution of Possible, Probable, or Definite |  |
| **GRADES 4 AND 5**  Regardless of Attribution |  | **GRADES 4 AND 5**  Regardless of Attribution |  |
| **Refer to Section 10.2.3 for detailed reporting requirements related to Important Medical Events (IME), Adverse Events of Special Interest (AESI), >30 days post treatment, and Pregnancy**. | | | |

Offline reporting when iMedidata Rave is unavailable:

If the iMedidata Rave system is unavailable to the site staff to report the SAE, the information is to be reported to Mayo Clinic by completing, scanning, and submitting a hard copy of the “Adverse Events” CRF and related “SAE: Event Information” CRFs within 24 hours of the investigator’s knowledge of the event. See the study forms packet for printable copy of these forms. Completed forms are to be submitted to [INDSafety@mayo.edu](mailto:INDSafety@mayo.edu).

For studies where the first notification of a SAE is reported to Mayo Clinic using the Offline reporting instructions, the data must be entered into iMedidata Rave when the system is again available. The originally submitted forms and the email correspondence must be kept within the study file at the study site.

10.2.2.3 Exceptions for Site(s) Regarding Expedited Reporting Timelines for SAEs

For this protocol only, the following AEs/Grades are expected to occur within this population and do not require Expedited Reporting. These events must still be reported via iMedidata Rave (see **Section 10.2.1.1**):

- - - - Specific protocol exceptions to expedited reporting should be reported immediately by investigators **ONLY** if they exceed the expected grade of the event.

| **CTCAE System Organ Class (SOC)** | **Adverse event/ Symptoms** | **CTCAE Grade at which the event will not be reported in an expedited manner^1^** |
| --- | --- | --- |
| General disorders and administrations site conditions | Fatigue | ≤Grade 3 |
|  | Pain | ≤Grade 3 |
|  | Dry eye | ≤Grade 3 |
| Skin and subcutaneous tissue disorders | Alopecia | ≤Grade 2 |
|  | Mucositis oral | ≤Grade 3 |
| Gastrointestinal disorders (GI) | Vomiting | ≤Grade 3 |
|  | Esophagitis | ≤Grade 3 |
|  | Dysphagia | ≤Grade 3 |
|  | Constipation | ≤Grade 3 |
|  | Nausea | ≤Grade 3 |
|  | Diarrhea | ≤Grade 3 |
|  | Dry mouth | ≤Grade 4 |
| Injury, poisoning and procedural complications | Dermatitis radiation | ≤Grade 3 |

*These exceptions only apply if the AE does not result in hospitalization. If the AE results in hospitalization, then the standard expedited AEs reporting requirements must be followed. The following hospitalizations are not considered to be SAEs because there is no “AE” (i.e., there is no untoward medical occurrence) associated with the hospitalization:

- Hospitalizations for respite care.
- Planned hospitalizations required by the protocol.
- Hospitalization planned before informed consent (where the condition requiring the hospitalization has not changed post study intervention).
- Hospitalization for elective procedures unrelated to the current disease and/or treatment on this trial.
- Hospitalization for administration of study intervention.
- Hospitalization for routine maintenance of a device (e.g., battery replacement) that was in place before study entry.
- Hospitalization or other serious outcomes for signs and symptoms of progression of the cancer.

#### 10.2.3 Other Reporting

10.2.3.1 >30 Days Post Treatment

Immediately, upon study team’s awareness of event, submit initial and follow-up (as applicable) reporting as follows:

- - SAEs that occur > 30 days after the last administration of investigational agent/intervention and have an attribution of possible, probable, or definite should be reported to Mayo Clinic (refer to **Section 10.2.2**).
  - For AEs occurring >30 days after the last administration of investigational agent/intervention, refer to **Section 10.2.1.1** for reporting requirements.

10.2.3.2 Pregnancy and Pregnant Partner

If a subject of childbearing potential becomes pregnant, or a subject of reproductive potential fathers a child, while the subject is on protocol, report the pregnancy to Mayo Clinic as specified below.

NOTES: Pregnancies and/or lactations that occur after registration but before treatment allocation/randomization must be reported by the investigator if they cause the subject to be ineligible from continued participation on the trial.

Pregnancies and lactations that occur from the time of treatment allocation/randomization through 30 days following cessation of study intervention, or 30 days following cessation of treatment if the subject initiates new anticancer therapy, whichever is earlier, must be reported by the investigator.

10.2.3.2.1 Pregnancy

Immediately, upon the Investigator becoming aware of a pregnancy, the site must contact [INDSafety@mayo.edu](mailto:INDSafety@mayo.edu) for reporting instructions. Mayo Clinic Comprehensive Cancer Center FDA Coordination Team will follow-up with the investigator regarding additional information that may be required.

If any person becomes pregnant during the study, the investigator should attempt to obtain information regarding the birth outcome and health of the infant. If the outcome of the pregnancy meets a criterion for immediate classification as a Serious Adverse Event (i.e., a person of childbearing potential experiences a spontaneous abortion, stillbirth, or neonatal death or there is a fetal or neonatal congenital anomaly) the investigator will report the event as a Serious Adverse Event.

Pregnancy must be reported in an expedited manner as **Grade 3 “Pregnancy, puerperium and perinatal conditions - Other (pregnancy)”** under the Pregnancy, puerperium and perinatal conditions System Organ Class (SOC). Pregnancy should be followed until the outcome is known.

10.2.3.2.2 Pregnant Partner

If a subject’s partner becomes pregnant, the investigator should discuss obtaining information regarding the birth outcome and health of the infant from the pregnant partner. **Prior to obtaining private information about a childbearing person and their infant, the Investigator must obtain consent from the childbearing person and the newborn infant’s parent or legal guardian before any data collection can occur. A consent form will need to be submitted to the IRB for these subjects if a pregnancy occurs.**

NOTE: If informed consent is not obtained, no information may be collected.

The site must contact [INDSafety@mayo.edu](mailto:INDSafety@mayo.edu) for reporting instructions. Mayo Clinic Cancer Center FDA Coordination Team will follow-up with the investigator regarding additional information that may be required.

10.2.3.3 Death

A death occurring between the time of registration and ≤30 days after last dose of study treatment requires iMedidata Rave reporting regardless of causality. Attribution to treatment or other cause must be provided.

Reportable categories of Death

- Death Not Otherwise Specified (NOS): A cessation of life that cannot be attributed to a CTCAE term associated with Grade 5.
- Sudden death NOS: A sudden (defined as instant or within one hour of the onset of symptoms) or an unobserved cessation of life that cannot be attributed to a CTCAE term associated with Grade 5.
- Death due to progressive disease that cannot be attributed to a CTCAE term associated with Grade 5 should be reported as **Grade 5 “Disease progression” under the SOC of “General disorders and administration site conditions.”** Evidence that the death was a manifestation of underlying disease (e.g., radiological changes suggesting tumor growth or progression: clinical deterioration associated with a disease process) should be submitted.
- All other causes of “Death” must be attributable to a Grade 5 CTCAE term.
- Death will only be expeditiously reported if it occurs ≤ 30 days after the last study dose of radiation AND the death is deemed possibly, probably or definitely related to the radiation treatment by treating provider and/or site PI. All other deaths will be reported during continuing review as part of the ongoing AE log if they meet reportable criteria above.

10.2.3.4 Secondary Malignancy

- A secondary malignancy is a cancer caused by treatment for a previous malignancy (e.g., treatment with investigational agent/intervention, radiation or chemotherapy). A secondary malignancy is not considered a metastasis of the initial neoplasm.
- All secondary malignancies that occur following treatment with an agent under an IND will be reported. Three options are available to describe the event:
- Leukemia secondary to oncology chemotherapy (e.g., Acute Myelocytic Leukemia [AML])
- Myelodysplastic syndrome (MDS)
- Treatment-related secondary malignancy
  - Any malignancy possibly related to cancer treatment (including AML/MDS) should also be reported via iMedidata Rave.

10.2.3.5 Second Malignancy

A second malignancy is one unrelated to the treatment of a prior malignancy (and is NOT a metastasis from the initial malignancy). Second malignancies require ONLY routine reporting via iMedidata Rave unless otherwise specified.

10.2.3.6 Protocol Violation/Deviation Reporting

The Mayo Clinic [INDSafety@mayo.edu](mailto:INDSafety@mayo.edu) inbox must be notified immediately, of any Major Deviation (defined in **Section 10.1.6**).

**Mayo Clinic Sites:** All Major and Minor deviations must be entered in PTrax immediately upon the study team becoming aware of the violation/deviation. Mayo Clinic staff are responsible for reporting to the Mayo Clinic IRB per IRB policy.

**External NON-Mayo Clinic Sites:** All deviations must be entered into the appropriate iMedidata Rave eCRF. Non-Mayo Clinic Sites are responsible to report to their local IRB per IRB policy.

### 10.3 Sponsor-Investigator Reporting

Per FDA, 21 CFR 312.32, Mayo Clinic will report SAEs and/or suspected unexpected serious adverse reactions as required to regulatory authorities, investigators/institutions, and IRBs/IECs in compliance with all reporting requirements according to local regulations and Good Clinical Practice.

Mayo Clinic Cancer Center (MCCC) FDA Coordination Team will assist the sponsor-investigator in the processing of expedited reports (i.e., IND Safety reports, deviations, dosing errors, pregnancy, etc.), as appropriate.

The MCCC FDA Coordination Team will report SAEs to the pharma partner(s) per contract.

## **11.0 Treatment Evaluation/Measurement of Effect**

See Appendix III: Criteria for Response Assessment

11.1 Patients will be evaluated at baseline, then according to the Assessment Schedule (**Section 4.0**, toxicity assessment, adverse event collection, PET/CT evaluation per Lugano criteria, survival, etc.)

11.2 At the time of reevaluation, patients will be classified in the following manner:

- No evidence of disease
- Recurrence of disease. Consider biopsy of the site, salvage systemic therapy, etc. per treatment team discretion.

11.3 The radiographic site of progression (or failure) will also be collected and classified as either:

- Local: centered within the 95% isodose line of the treatment plan.
- Distant: outside the 95% isodose line of the treatment plan.

11.4 Response assessment

Schedule of evaluations: All patients are required to receive a PET/CT scan at baseline. Post-treatment tumor recurrence will be monitored with follow-up PET/ CT scans to assess tumor response based on Lugano criteria until local progression within radiation field or death (or up to 24 months after the completion of radiation treatment) at which point participants will go to survival follow-up. Patients who did not test FDG negative at baseline are strongly recommended to receive a PET/CT or CT if PET/CT is not done, 3 months after treatment as part of post-treatment follow-up. Patients will receive a follow-up CT scan at 6 months post-treatment and will continue to do so every 6 months at clinician’s discretion. For patients without measurable disease on baseline PET/CT or CT (eg cutaneous lymphoma) follow up imaging is not required and response will be based on clinical exam.

Patients with gastric lymphoma will receive an endoscopy prior to registration. Post-treatment tumor recurrence will be monitored with follow-up endoscopies at 3 months and every 6 months per clinician’s discretion after treatment.

The Lugano criteria is outlined in Appendix III. For gastric lymphoma, additional information regarding pathologic response will be provided via endoscopy.


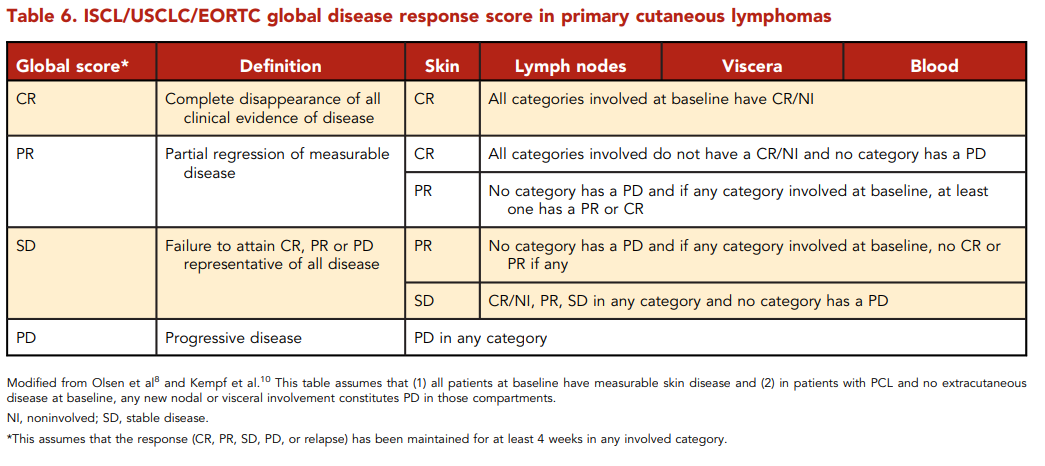


## Table from Olsen *et al* see reference 17.

## **12.0 Descriptive Factors**

• Age (≤60 years and >60 years)

• Sex (M/F/Unknown/Intersex)

• Diagnosis/Histology (Follicular I/II, Follicular IIIA, Marginal Zone/Malt, Other)

• Reason for radiotherapy (curative/palliative)

• Eastern Cooperative Oncology Group (ECOG) performance status (0, 1, 2 vs. 3)

• Ann Arbor stage (Stage I/II vs. III/IV/relapse/refractory)

• Target site maximum diameter (>6 cm vs. ≤6 cm)

• Lactate dehydrogenase at registration/first treatment (≤ULN vs. >ULN)

• Extranodal site (yes/no)

• Previous radiation therapy (yes/no)

• Previous chemotherapy (yes/no)

• Number of sites treated in this trial (≤2 or >2)

## **13.0 Treatment/Follow-up Decision at Evaluation of Patient**

13.1 Those patients who will not receive any radiation treatment or who will receive radiation treatment elsewhere will go off study per **Section 13.7**.

13.2 Patient who are CR, PR, or SD will continue to obtain PET or CT scans at each clinically indicated follow up for up to 24 months after the completion of radiation treatment.

13.3 Patients who develop PD or withdraw from further follow up assessments will be followed for event monitoring only per **Section 4.0**.

13.4 Inevaluable patients

If a patient fails to complete Radiation Therapy for reasons other than toxicity, the patient will be regarded as unevaluable.

### 13.5 Ineligible

A patient is deemed *ineligible* if after registration, it is determined that at the time of registration, the patient did not satisfy each and every eligibility criterion for study entry. If the patient receives treatment, the patient may continue treatment at the discretion of the physician as long as there are not safety concerns. The patient will continue in the Active Monitoring/Treatment phase of the study, as per **Section 4.0** of the protocol.

- - If the patient never received treatment, on-study material must be submitted. Survival Follow-up will be required per **Section 4.1** of the protocol.

### 13.6 Major Violation

A patient is deemed a *major violation*, if protocol requirements regarding treatment in cycle 1 of the initial therapy are severely violated that evaluability for primary end point is questionable. If the patient receives treatment, the patient may continue treatment at the discretion of the physician as long as there are no safety concerns. The patient will continue in the Active Monitoring/Treatment phase of the study, as per **Section 4.0** of the protocol.

### 13.7 Cancel

A patient is deemed a *cancel* if he/she is removed from the study for any reason before any study treatment is given. On-study material and the End of Protocol Treatment/Intervention must be submitted. No further data submission is necessary.

## **14.0 Body Fluid Biospecimens**

None

## **15.0 Drug/Device Information**

None

## **16.0** **Statistical Considerations and Methodology**

16.1 Study Design

The study is designed as a two arm 1:1 randomized Phase II trial with a total evaluable sample size of 102 patients (51 patients per arm) to evaluate an intermediate dose of 9 Gy (delivered over 3 fractions) or similar dose as described versus the standard of care (SOC) 24 Gy in 12 fractions for patients with indolent lymphoma, while simultaneously evaluating prognostic value of pre-radiation functional imaging parameters and molecular characteristics to predict treatment response and failure.

### 16.2 Primary endpoints

The following definition is used for the primary endpoint of interest:

- Acute toxicity: any adverse event grade 2 or higher (CTCAE v5.0) possibly related to radiation treatment, reported at End of Treatment, Day 7 post RT, or Day 14 post RT. Acute toxicity is reported as a proportion calculated as the number of patients with acute toxicity divided by the total number of treated patients.

### 16.3 Secondary

The following definitions are used for the secondary endpoints of interest:

• Response rate at 3 months post radiation treatment: is reported as a proportion calculated as the number of cases with complete response (CR) or a partial response (PR) at 3-month post radiation imaging based on Lugano criteria divided by the total number of treated patients. Treated patients who do not have a 3-month evaluation will be classified as a non-response.

• Local control rate at 2-years: defined from the time of registration to local relapse that overlaps the treated site (95% isodose line).

• Patient Reported Outcomes/Quality of life: FACIT-Fatigue given as SOC for Lymphoma patients within 3 months after end of radiation treatment will be used as outlined in section 4.0 and 7.0.

16.4 Exploratory endpoints

16.4.1 Archived tissue collected from biopsy of enrolled patients will be sent for pathological assessment of cellular and genetic mutations to correlate them with disease local relapse and radiation resistance, where applicable. This includes:

16.4.1.1 TP53 gene alteration by Fluorescence in situ hybridization (FISH)

16.4.1.2 Expression of TP53, MYC and proliferation index (Ki-67) by immunohistochemistry stain (IHC)

16.4.2 Baseline PET/CT scan of patients will be sent to MIMvista to undergo autosegmentation to calculate the MTV, TLG and SUVmax of their disease to correlate it with disease local relapse and treatment response.

16.4.3 Financial toxicity: financial side effects induced by cancer treatment that will be assessed by a modified COST-FACIT questionnaire as outlined in section 4.0 and 7.0.

16.4.4 Financial health care expenditure reflects the costs spent on health care and related activities attributable to this cancer treatment and will be assessed as outlined in section 4.0 and 7.0.

16.4.5 Sensitivity analysis to assess differences in the different radiation dose regimens in the experimental treatment arm.

### 16.5 Statistical Design

16.5.1 Decision Rules

16.5.1.1 A two group χ^2^ test of equal proportions in acute adverse events will be conducted to determine the efficacy in acute toxicity reduction within the treatment arm. If the p-value of the statistical test is less than 0.10 then the treatments will be considered significantly different in the direction of the point estimates. Therefore, a significant difference in the direction of the experimental arm will deem the treatment favorable and worth consideration in future trials. If the test is non-significant then the experimental arm will not be considered significantly different in toxicity profile and therefore, not considered for future trials. It is also important to consider the local control rate when deciding whether to pursue further study. In the FoRT trial, the 2-year local progression-free rate was 94.1% after 24 Gy and 79.8% after 4 Gy. If the observed 2-year probability of local control is more than 7% inferior with 9 Gy versus 24 Gy, then we will not consider future trials. For example, if the 2-year local control rate is 94.1% after 24 Gy, and less than 87.1% after 9 Gy then we would not pursue further study.

16.5.1.2 Over Accrual: We will accrue approximately 10% over-accrual (112 total patients, 56 per arm) in order to ensure 102 evaluable patients as described in previous sections. The first 102 evaluable patients will be utilized for analysis of the primary endpoint.

16.5.2 Sample Size

102 evaluable patients will be accrued to this phase-II study unless undue toxicity is encountered. We anticipate accruing an additional 10 patients to account for ineligibility, cancellation, major treatment violation, or other reasons. Maximum projected accrual is therefore 112 patients.

16.5.3 Accrual Time and Study Duration

We anticipate enrolling 112 patients and thus plan to finish accrual of patients in 1.5-2 years after opening the study. Therefore, the overall study duration is expected to be 48 months.

16.5.4 Power and Significance Levels

The study is designed as a two arm 1:1 randomized Phase II trial with a total evaluable sample size of 102 patients and a 1:1 randomization (51 patients per arm). We will utilize a two-group chi-squared test of equal proportions with a two-sided α = 0.10 to test the primary hypothesis given acute toxicity estimates observed in the FoRT trial (grade 2+ acute toxicity of approximately 31% in the 24 Gy arm and approximately 9% in the 4 Gy arm). We hypothesize similar estimates in the 24 Gy arm, and we hypothesize acute toxicity rates of 10% in the 9 Gy arm. With a two-sided alpha of 0.10 we will achieve 80% power to detect difference in proportions of 0.21 when the acute toxicity is assumed to be 0.31 in the standard of care arm and 0.10 in the experimental arm. To ensure evaluability of our primary endpoint we will accrue 10 additional patients (56 per arm) to account for approximately 10% lost to follow-up.

16.5.5 Other Considerations

Adverse events observed in this study as well as scientific discoveries or changes in standard care will be taken into account in any decision to terminate the study.

16.6 Analysis Plan

The primary analysis for this trial will commence once the first 102 evaluable patients have completed their Day 14 Post ISRT visit. It is anticipated that the earliest date in which the results will be made available via a manuscript, abstract, or presentation format is when 102 patients have experienced an acute toxicity or 2 weeks post radiation therapy follow-up.

16.6.1 Primary Analysis: The primary analysis will be to compare the proportion of patients who experience an acute toxicity within the first 2 weeks of finishing RT between the 2 treatment arms.

16.6.1.1 Acute toxicity: All patients who were registered to the study and started treatment will be included in the acute toxicity analysis. Acute toxicity has been defined in section 16.2. A chi-squared test will be used to compare acute toxicity rates between treatment arms. A two-sided p-value less than 0.10 will be considered statistically significant (see decision rule). To assess the size of differences, absolute risk difference and relative risk will be calculated; 95% confidence intervals will be calculated using the score method. Although we will use an alpha of 0.10 for deciding whether to pursue further study, 95% confidence intervals will be reported for estimates for ease of interpretation.

16.6.2 Secondary Analyses

16.6.2.1 Response Rate: All patients who were registered to the study and started treatment will be included in the response rate analysis. Response rate will be defined as the proportion of patients displaying response (PR or CR) at 3 months post treatment; patients who did not have a 3-month post treatment assessment will be considered a non-response. A chi-square test will be used to compare response rates between the two treatment arms. Absolute risk difference and relative risk will be calculated; 95% confidence intervals will be calculated using the score method.

16.6.2.2 Local control: All patients who were registered to the study and started treatment will be included in the local control analysis. A local recurrence event is any event defined as disease relapse occurring in the area treated before with radiation (anything included within the 95% isodose line). The local control interval will be defined as the number of days from end of radiation treatment until first local recurrence within 24 months post radiation treatment; censoring will occur at date of patient death or date of last disease evaluation (based on imaging and clinical exam). Local control rates will be estimated separately for the two treatment groups at 3-, 6-, 12-, 18, and 24-months post radiation treatment using the Kaplan-Meier method. Hazard ratio and 95% confidence interval for comparison of local recurrence between the two treatment arms will be estimated from a Cox proportional hazards model.

16.6.2.4 Quality of life: FACIT-Fatigue scores along with validated subscales given as SOC for Lymphoma patients will be collected from baseline to up to 3 months after radiation treatment. The FACIT Fatigue consists of 13 Likert-scale items scored 0 (not at all) to 4 (very much). We will calculate the fatigue subscale score at each assessment by first reverse scoring the following items: HI7, HI12, An1, An2, An3, An4, An8, An12, An14, An15, An16 by subtracting the response from 4 and then sum the 13 items to obtain a score. Then multiple the sum of the items by the number of items in the subscale (13), then divide by the number of items the patient answered. The Fatigue Subscale score ranges from 0 to 52 where a higher score indicates a better quality of life. We will estimate the difference between treatment arms in the trajectory (slope) of the Fatigue Subscale score including all available scores from end of treatment to 3 months post RT in a longitudinal mixed effects regression model with a random effect for patient. Fixed effects will include an indicator variable for treatment arm (1=9 Gy, 0=24 Gy), time defined as the number of weeks (7 days) from end of treatment to FACIT-Fatigue assessment (continuous), and an interaction term for treatment arm and time. The estimates from the interaction term along with 95% confidence interval (profile likelihood) will be interpreted as the difference in trajectories between treatment arms. A likelihood ratio test will be used to compare treatment arms on the difference in trajectories of fatigue subscale score.

16.6.2.5 Exploratory Analyses

Financial toxicity: COST-FACIT with added questions to assess specifically for travel and accommodation expenses, travel distance and time, and loss of work time for patient and care givers scores will be assessed at end of treatment and described separately for the two groups. The financial toxicity score will be calculated using items FT1-FT11 where items FT2, FT3, FT4, FT5, FT8, FT9, and FT10 will first need to be reverse scored by subtracting the response from 4 and then sum the individual items to obtain a score. Then multiply the sum of the item scores by the number of items in the scale (11) and divide by the number of items the patient answered. The financial toxicity score ranges from 0-44 where a higher score indicated better financial well-being. The financial toxicity score will be summarized separately for the two treatment groups with the mean, standard deviation, median, 25^th^ percentile, and 75^th^ percentile. No comparison between treatment groups will be conducted. Individual items will be described with mean, standard deviation.

Financial health care expenditure will be determined by first obtaining base cost rates from Mayo Clinic’s reimbursement database for all codes included in the planning, treatment, and management of patient’s enrolled in both arms. Mean total radiotherapy costs will then be calculated using inflation adjusted standardized Medicare rates and summarized separately for the 2 treatment arms; a linear regression model will be used to estimate the difference in Financial health care expenditure between the two treatment arms.

Biopsies of enrolled patients will be evaluated for pathological assessment of cellular and genetic mutations. This includes: TP53 alteration by FISH and expression of TP53, MYC and proliferation index (Ki-67) by immunohistochemistry stain (IHC).

Baseline PET/CT scan of patients will be sent to MIMvista to undergo autosegmentation to calculate the MTV, TLG and SUVmax of their disease.

Multivariable models will be used to evaluate associations of these biomarkers separately with local control (logistic regression) and response (Cox proportional hazards). Models will include biomarker, treatment arm as a covariate along with potentially confounding variables.

### 16.7 Data and Safety Monitoring

#### 16.7.1 Safety Review

The principal investigators and the study statistician will review the study monthly to identify accrual, adverse event, and any endpoint problems that might be developing. The Mayo Clinic Comprehensive Cancer Center (MCCCC) Data Safety Monitoring Board (DSMB) is responsible for reviewing accrual and safety data for this trial at least biannually, based on reports provided by the MCCCC Statistical Office.

#### 16.7.2 Adverse Event Stopping Rules

The stopping rules specified below are based on knowledge available at study development. We note that the Adverse Event Stopping Rule may be adjusted in the event of either (1) the study re-opening to accrual or (2) at any time during the conduct of the trial and in consideration of newly acquired information regarding the adverse event profile of the treatment(s) under investigation. The study team may choose to suspend accrual because of unexpected adverse event profiles that have not crossed the specified rule below.

Accrual will be temporarily suspended if at any time we observe events considered at least possibly related to study treatment (i.e., an adverse event with attribute specified as “possible,” “probable,” or “definite”) that satisfy one of the following:

• If 4 or more patients in the first 10 treated patients (per each treatment regimen; 1. 10 Gy in 5 fractions, 2. 8 Gy in 2 fractions, 3. 9 Gy in 3 fractions, 4. 24 Gy in 12 fractions (i.e., SOC)) experience a grade 3 or higher adverse event, at least possibly related to treatment at any time following completion of the protocol treatment within 90 days post-treatment.

• After the first 10 patients have been treated (per treatment arm): if ≥ 40% of all patients experience a grade 3 or higher adverse event, at least possibly related to treatment following the completion of protocol treatment to 90 days post-treatment.

We note that we will review grade 4 and 5 adverse events deemed “unrelated” or “unlikely to be related”, to verify their attribution and to monitor the emergence of a previously unrecognized treatment-related adverse event.

### 16.8 Subset Analyses for Minorities

#### 16.8.1 Study Availability

This study will be available to all eligible patients, regardless of gender, race, or ethnic origin.

#### 16.8.2 Statistical Analysis by Subset

There is no information currently available regarding differential effects of this regimen in subsets defined by race, gender, or ethnicity, and there is no reason to expect such differences to exist. Therefore, although the planned analyses will look for differences in treatment effect based on racial groupings, the sample size is not increased to provide additional power for subset analyses.

16.8.3 Regional Population

The geographical region served by MCCCC has a population which includes 3% minorities. Expected sizes of racial by gender subsets are shown in the following table:

| **Accrual Targets** | | | |
| --- | --- | --- | --- |
| **Ethnic Category** | **Sex/Gender** | | |
|  | **Females** | **Males** | **Total** |
| Not Hispanic or Latino | 42 | 42 | 84 |
| Hispanic or Latino | 14 | 14 | 28 |
| **Ethnic Category: Total of all subjects** | 56 | 56 | 112 |
|  | | | |
| American Indian or Alaskan Native | 1 | 1 | 2 |
| Asian | 1 | 1 | 2 |
| Black or African American | 11 | 11 | 22 |
| Native Hawaiian or other Pacific Islander | 0 | 0 | 0 |
| White (Hispanic and non-Hispanic) | 43 | 43 | 86 |
| **Racial Category: Total of all subjects** | 56 | 56 | 112 |

| **Ethnic Categories:** | **Hispanic or Latino** **–** A person of Cuban, Mexican, Puerto Rican, South or Central American, or other Spanish culture or origin, regardless of race. The term “Spanish origin” can also be used in addition to “Hispanic or Latino”  **Not Hispanic or Latino** |
| --- | --- |
| **Racial Categories:** | **American Indian or Alaskan Native** **–** A person having origins in any of the original peoples of North, Central, or South America, and who maintains tribal affiliations or community attachment.  **Asian** **–** A person having origins in any of the original peoples of the Far East, Southeast Asia, or the Indian subcontinent including, for example, Cambodia, China, India, Japan, Korea, Malaysia, Pakistan, the Philippine Islands, Thailand, and Vietnam. (Note: Individuals from the Philippine Islands have been recorded as Pacific Islanders in previous data collection strategies.  **Black or African American** **–** A person having origins in any of the black racial groups of Africa.  **Native Hawaiian or other Pacific Islander** **–** A person having origins in any of the original peoples of Hawaii, Guam, Samoa, or other Pacific Islands.  **White** **–** A person having origins in any of the original peoples of Europe, the Middle East, or North Africa. |

## **17.0 Pathology Considerations/Tissue Biospecimens**

17.1 Biospecimens Table

| **Correlative Study (See Section 17.3 for more information)** | **Mandatory or Optional** | **Type of Tissue to Collect** | **Block, Slides, Core, etc. (# of each to submit)** | **Biopsy** | **Process at site? (Yes or No)** | **Temperature**  **Conditions for Storage /Shipping** |
| --- | --- | --- | --- | --- | --- | --- |
| Correlative Histopathologic Research  (see below)-TP53 | Optional^1^ | Formalin-fixed paraffin embedded tissue | Formalin-fixed paraffin embedded (FFPE)  block (1) | X | Yes | Room temperature |
| Correlative Histopathologic Research  (see below)-Ki-67 | Optional^1^ | Formalin-fixed paraffin embedded tissue | FFPE block (1) | X | Yes | Room temperature |
| Correlative Histopathologic Research  (see below)-MYC | Optional^1^ | Formalin-fixed paraffin embedded tissue | FFPE block (1) | X | Yes | Room temperature |

^1^*If enough tissue is present for analysis after standard clinical pathological evaluation*

17.2 Diagnostic Slides from Original and /or Recurrent Tissue

N/A

17.3 Correlative Tissue Collection

17.3.1 Correlative tissue collection will occur only if enough tissue is present for analysis after standard clinical pathological evaluation.

17.3.2 Formalin-fixed paraffin embedded tissue

17.3.2.1 Formalin-fixed paraffin embed is the standard process used for clinical pathology evaluation after the tissue is biopsy.

17.3.2.2 Patients will be consented before biopsy if the pathology assessment happens at our institute. After the final pathology diagnosis is rendered and enough tissue is left on the paraffin block, Dr. Jennifer Jiang (the pointed pathologist) will request the correlative studies.

17.3.2.3 If the diagnosis was rendered at outside institutions, we will attempt to request appropriate FFPE material from outside institutes.

17.4 Background and Methodology

17.4.1 *TP53*

*TP53* is associated with more aggressive disease.

Tissue samples will be collected for assessment of *TP53* gene alteration by Fluorescence in situ hybridization (FISH) and expression by immunohistochemistry stain (IHC) in order to study its correlation with disease progression and radiation resistance.

17.4.2 Proliferation index (Ki-67)

Proliferation index (Ki-67) role with radiation sensitivity and treatment outcome is controversial.

Tissue samples will be collected for assessment of proliferation index (Ki-67) by IHC to study its correlation with disease progression and radiation resistance.

17.4.3 *MYC*

*MYC* expression has been associated with more aggressive disease.

Tissue samples will be collected for assessment MYC expression by IHC to study its correlation with disease progression and radiation resistance.

## **18.0 Records and Data Collection Procedures**

### 18.1 Submission Timetable

Data submission instructions for this study can be found in the Data Submission Schedule.

### 18.2 Survival Follow-up

See **Section 4.0**

### 18.3 CRF Completion

This study will use Medidata Rave^®^ for remote data capture (rdc) of all study data. Data collection for this study will be done exclusively through the Medidata Rave^®^ clinical data management system. Access to the trial in Rave is granted through the iMedidata application to all persons with the appropriate roles assigned in Regulatory Support System (RSS). To access Rave via iMedidata, the site user must have an active account and the appropriate Rave role (Rave CRA, Read-Only, Site Investigator) on the organization roster at the enrolling site.

### 18.4 Supporting Documentation

This study requires supporting documentation for diagnosis and progression prior to study entry as well as for evidence of response to study therapy and progression after study therapy and includes the tumor tissue biopsy pathology report, bone marrow biopsy and aspirate report, and imaging report(s). These documents should be submitted within 14 days of registration (for prior to study entry materials) or within 14 days after the visit at which response or progression is determined.

### 18.5 Labeling of Materials

Each site will be responsible for ensuring that all materials contain the patient’s initials, MCCCC Registration Number, and MCCCC protocol number. The patient’s name must be removed.

### 18.6 Overdue Lists

A list of overdue forms and outstanding queries will be available in Rave through the Rave Task Summary. In addition to this, the Overdue Materials report will be distributed monthly by the data manager.

## **19.0 Budget**

### 19.1 Costs Charged to Patient: Routine clinical care

### 19.2 Tests to be Research Funded: Pathological and molecular assessment of the tissue samples as outlined above, and baseline imaging auto segmentation.

### **20.0 References**

1. Swerdlow, S.H., et al., eds. WHO Classification of Tumours of Haematopoietic and Lymphoid Tissues. ed. th. 2008, WHO Press: Geneva, Switzerland.

2. Mac Manus MP, Hoppe RT. Is radiotherapy curative for stage I and II low-grade follicular lymphoma? Results of a long-term follow-up study of patients treated at Stanford University. J Clin Oncol. 1996;14(4):1282-1290.

3. Gospodarowicz MK, Bush RS, Brown TC, Chua T. Prognostic factors in nodular lymphomas: a multivariate analysis based on the Princess Margaret Hospital experience. Int J Radiat Oncol Biol Phys. 1984;10(4):489- 497.

4. Vaughan Hudson B, Vaughan Hudson G, MacLennan KA, Anderson L, Linch DC. Clinical stage 1 nonHodgkin's lymphoma: long-term follow-up of patients treated by the British National Lymphoma Investigation with radiotherapy alone as initial therapy. Br J Cancer. 1994;69(6):1088-1093.

5. Pugh TJ, Ballonoff A, Newman F, Rabinovitch R. Improved survival in patients with early stage low-grade follicular lymphoma treated with radiation: a Surveillance, Epidemiology, and End Results database analysis. Cancer. 2010;116(16):3843-3851.

6. Lowry L, Smith P, Qian W, et al. Reduced dose radiotherapy for local control in non-Hodgkin lymphoma: a randomised phase III trial. Radiother Oncol. 2011;100(1):86-92.

7. Campbell BA, Voss N, Woods R, et al. Long-term outcomes for patients with limited stage follicular lymphoma: involved regional radiotherapy versus involved node radiotherapy. Cancer. 2010;116(16):3797- 3806.

8. Pinnix, Chelsea C et al. Ultra-low-dose radiotherapy for definitive management of ocular adnexal B-cell lymphoma. Head & neck vol. 39,6 (2017): 1095-1100. doi:10.1002/hed.24717.

9. Hoskin P et al. 4 Gy versus 24 Gy radiotherapy for follicular and marginal zone lymphoma (FoRT): long-term follow-up of a multicentre, randomised, phase 3, non-inferiority trial. Lancet Oncol. 2021 Mar;22(3):332-340. doi: 10.1016/S1470-2045(20)30686-0. Epub 2021 Feb 1. PMID: 33539729.

10. Delfau-Larue, M.H., et al., Total metabolic tumor volume, circulating tumor cells, cell-free DNA: distinct prognostic value in follicular lymphoma. Blood Adv, 2018. 2(7): p. 807-816.

11. Wong, W.L., et al., Evaluation of normal FDG uptake in palatine tonsil and its potential value for detecting occult head and neck cancers: a PET CT study. Nucl Med Commun, 2007. 28(9): p. 675-80.

12. Meignan, M., et al., Baseline Metabolic Tumor Volume Predicts Outcome in High-Tumor-Burden Follicular Lymphoma: A Pooled Analysis of Three Multicenter Studies. J Clin Oncol, 2016. 34(30): p. 3618-3626.

13. Chatzitolios, A., et al., Prognostic significance of CD95, P53, and BCL2 expression in extranodal non-Hodgkin's lymphoma. Ann Hematol, 2010. 89(9): p. 889-96.

14. Aukema, S.M., et al., MYC expression and translocation analyses in low-grade and transformed follicular lymphoma. Histopathology, 2017. 71(6): p. 960-971.

15. O'Shea, D., et al., The presence of TP53 mutation at diagnosis of follicular lymphoma identifies a high-risk group of patients with shortened time to disease progression and poorer overall survival. Blood, 2008. 112(8): p. 3126-9.

16. Pocock, S. J., and R. Simon. 1975. "Sequential treatment assignment with balancing for prognostic factors in the controlled clinical trial." *Biometrics* 31 (1):103-15.

# 17. Olsen, E, et al, Primary cutaneous lymphoma: recommendations for clinical trial design and staging update from the ISCL,USCLC, and EORTC. Blood, 2022. 140(5): p.430.

# Appendix I: ECOG Performance Status

| **ECOG PERFORMANCE STATUS*** | |
| --- | --- |
| **Grade** | **ECOG** |
| 0 | Fully active, able to carry on all pre-disease performance without restriction |
| 1 | Restricted in physically strenuous activity but ambulatory and able to carry out work of a light or sedentary nature, e.g., light house work, office work |
| 2 | Ambulatory and capable of all selfcare but unable to carry out any work activities. Up and about more than 50% of waking hours |
| 3 | Capable of only limited selfcare, confined to bed or chair more than 50% of waking hours. |
| 4 | Completely disabled. Cannot carry on any selfcare. Totally confined to bed or chair. |
| 5 | Dead |

* As published in Am. J. Clin. Oncol.:
*Oken, M.M., Creech, R.H., Tormey, D.C., Horton, J., Davis, T.E., McFadden, E.T., Carbone, P.P.: Toxicity And Response Criteria Of The Eastern Cooperative Oncology Group. Am J Clin Oncol 5:649-655, 1982.*

The ECOG Performance Status is in the public domain therefore available for public use. To duplicate the scale, please cite the reference above and credit the Eastern Cooperative Oncology Group, Robert Comis M.D., Group Chair.

From <http://www.ecog.org/general/perf_stat.html>

# Appendix II: Patient Questionnaires

COST – FACIT (Version 2)

Below is a list of statements that other people with your illness have said are important**. Please circle or mark one number per line to indicate your response as it applies to the past 7 days.**

|  |  | **Not at all** | **A little bit** | **Some-what** | **Quite**  **a bit** | **Very much** |
| --- | --- | --- | --- | --- | --- | --- |
|  |  |  |  |  |  |  |
| FT1 | I know that I have enough money in savings, retirement, or assets to cover the costs of my treatment | 0 | 1 | 2 | 3 | 4 |
| FT2 | My out-of-pocket medical expenses are more than I thought they would be | 0 | 1 | 2 | 3 | 4 |
| FT3 | I worry about the financial problems I will have in the future as a result of my illness or treatment | 0 | 1 | 2 | 3 | 4 |
| FT4 | I feel I have no choice about the amount of money I spend on care | 0 | 1 | 2 | 3 | 4 |
| FT5 | I am frustrated that I cannot work or contribute as much as I usually do | 0 | 1 | 2 | 3 | 4 |
| FT6 | I am satisfied with my current financial situation | 0 | 1 | 2 | 3 | 4 |
| FT7 | I am able to meet my monthly expenses | 0 | 1 | 2 | 3 | 4 |
| FT8 | I feel financially stressed | 0 | 1 | 2 | 3 | 4 |
| FT9 | I am concerned about keeping my job and income, including work at home | 0 | 1 | 2 | 3 | 4 |
| FT10 | My cancer or treatment has reduced my satisfaction with my present financial situation | 0 | 1 | 2 | 3 | 4 |
| FT11 | I feel in control of my financial situation | 0 | 1 | 2 | 3 | 4 |
| FT12 | My illness has been a financial hardship to my family and me | 0 | 1 | 2 | 3 | 4 |

English (Universal) 26 September 2017

Copyright 2014, FACIT and The University of Chicago

PROMIS® Item Bank v1.0 – Fatigue – Short Form 13a (FACIT-Fatigue)

**Fatigue – Short Form 13a (FACIT-Fatigue)**

**Please respond to each question or statement by marking one box per row.**

|  | **During the past 7 days…** | **Not at all** | **A little bit** | **Somewhat** | **Quite a bit** | **Very much** |
| --- | --- | --- | --- | --- | --- | --- |
| HI7 | I feel fatigued | 🞎  1 | 🞎  2 | 🞎  3 | 🞎  4 | 🞎  5 |
|  |  |  |  |  |  |  |
| HI12 | I feel weak all over | 🞎  1 | 🞎  2 | 🞎  3 | 🞎  4 | 🞎  5 |
|  |  |  |  |  |  |  |
| AN1 | I feel listless ("washed out") | 🞎  1 | 🞎  2 | 🞎  3 | 🞎  4 | 🞎  5 |
|  |  |  |  |  |  |  |
| AN2 | I feel tired | 🞎  1 | 🞎  2 | 🞎  3 | 🞎  4 | 🞎  5 |
|  |  |  |  |  |  |  |
| AN3 | I have trouble starting things because I am tired | 🞎  1 | 🞎  2 | 🞎  3 | 🞎  4 | 🞎  5 |
|  |  |  |  |  |  |  |
| AN4 | I have trouble finishing things because I am tired | 🞎  1 | 🞎  2 | 🞎  3 | 🞎  4 | 🞎  5 |
|  |  |  |  |  |  |  |
| AN5 | I have energy | 🞎  5 | 🞎  4 | 🞎  3 | 🞎  2 | 🞎  1 |
|  |  |  |  |  |  |  |
| AN7 | I am able to do my usual activities | 🞎  5 | 🞎  4 | 🞎  3 | 🞎  2 | 🞎  1 |
|  |  |  |  |  |  |  |
| AN8 | I need to sleep during the day | 🞎  1 | 🞎  2 | 🞎  3 | 🞎  4 | 🞎  5 |
|  |  |  |  |  |  |  |
| AN12 | I am too tired to eat | 🞎  1 | 🞎  2 | 🞎  3 | 🞎  4 | 🞎  5 |
|  |  |  |  |  |  |  |
| AN14 | I need help doing my usual activities | 🞎  1 | 🞎  2 | 🞎  3 | 🞎  4 | 🞎  5 |
|  |  |  |  |  |  |  |
| AN15 | I am frustrated by being too tired to do the things I want to do | 🞎  1 | 🞎  2 | 🞎  3 | 🞎  4 | 🞎  5 |
|  |  |  |  |  |  |  |
| AN16 | I have to limit my social activity because I am tired | 🞎  1 | 🞎  2 | 🞎  3 | 🞎  4 | 🞎  5 |

English (Universal)

Copyright 1987, 1997 by David Cella, Ph.D.

© 2008-2023 PROMIS Health Organization (PHO)

# Appendix III: Criteria for Response Assessment


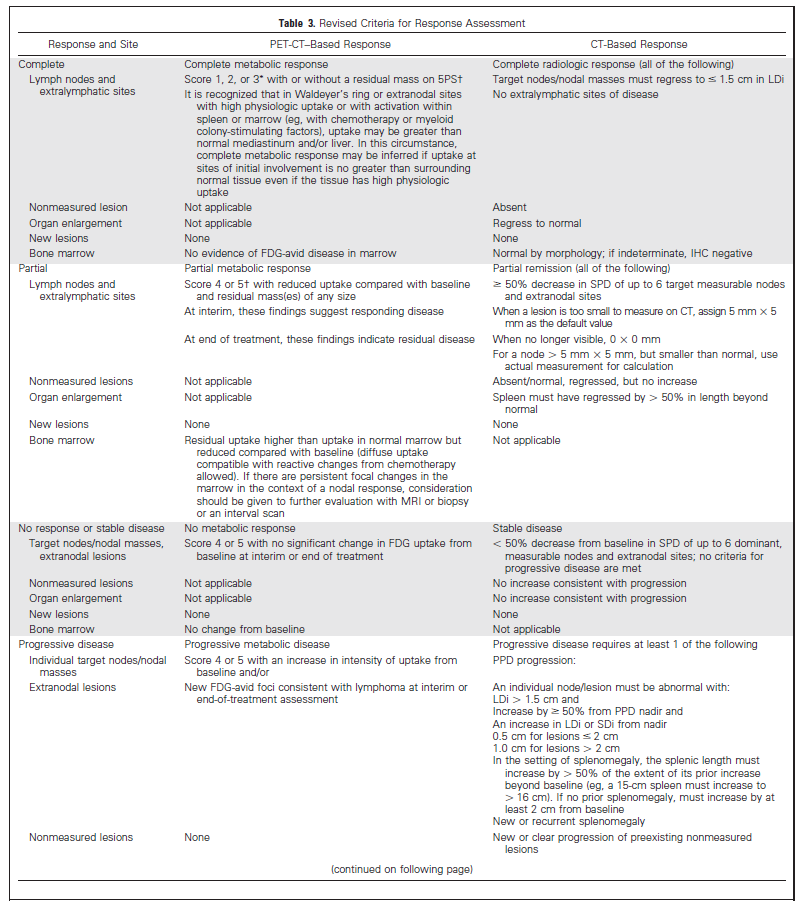


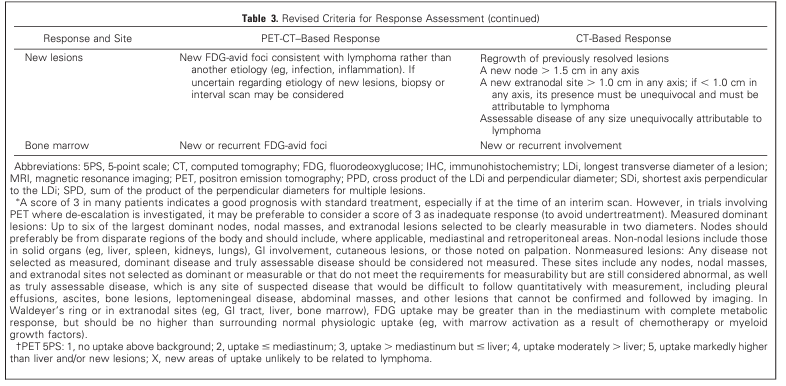

Supplement: MC230808_Protocol_MCCCCAmend#2_clean_17Jan2025 [file mmc1.docx]
